# Harnessing microbial allies: enhancing black alder resilience to PAH stress through microbial symbiosis

**DOI:** 10.3389/fpls.2025.1552258

**Published:** 2025-05-08

**Authors:** Greta Striganavičiūtė, Dorotėja Vaitiekūnaitė, Milana Šilanskienė, Vaida Sirgedaitė-Šėžienė

**Affiliations:** Laboratory of Forest Plant Biotechnology, Institute of Forestry, Lithuanian Research Centre for Agriculture and Forestry, Kaunas, Lithuania

**Keywords:** phytoremediation, microbial inoculants, plant-microbe interactions, biochemical plant responses, pollutants

## Abstract

Polycyclic aromatic hydrocarbons (PAHs) are persistent environmental pollutants that pose significant risks to plant health and ecosystem function. Phytoremediation, using plants in combination with microorganisms, offers a promising strategy for mitigating PAH toxicity. This study investigates the role of PAH-degrading microorganisms in mitigating the phytotoxic effects of PAHs on black alder (*Alnus glutinosa* L.) seedlings. Specifically, we examined the effects of three microbial strains—*Pseudomonas putida* Trevisan, *Sphingobium yanoikuyae* Yabuuchi et al., and *Rhodotorula* sp*haerocarpa* (S.Y. Newell & Fell) Q.M. Wang, F.Y. Bai, M. Groenewald & Boekhout—on plant growth and biochemical responses under exposure to naphthalene, pyrene, phenanthrene, and fluorene. The results revealed genotype-dependent variations in plant responses. In family 13-99-1K, *S. yanoikuyae* significantly enhanced defense mechanisms under phenanthrene exposure, evidenced by reduced malondialdehyde (MDA) levels and increased antioxidant enzyme activity. In contrast, family 41-65-7K exhibited stable shoot height and increased chlorophyll *a/b* ratio, but a decrease in soluble sugars under *P. putida* treatment with pyrene. This suggests a shift in metabolic priorities towards growth rather than stress mitigation. These findings highlight the complex interactions between plant genotype, microbial strain, and PAH type, underscoring the potential of microbial-assisted phytoremediation. Our study suggests that tailored microbial inoculants, in combination with appropriate plant genotypes, could optimize phytoremediation efforts in PAH-contaminated environments. Future research should focus on soil-based systems and longer-term evaluations to better understand the dynamics of plant-microbe-PAH interactions.

## 1 Introduction

Polycyclic aromatic hydrocarbons (PAHs) are a group of organic pollutants consisting of multiple fused aromatic rings (Sakshi et al., 2019). They are pervasive environmental contaminants originating primarily from incomplete combustion of organic materials, industrial activities, and fossil fuel usage (Sari et al., 2020). Their stability and hydrophobic nature make them highly persistent in soils, sediments, and aquatic environments, where they can pose significant health risks to both ecosystems and humans (Zhang et al., 2022). PAHs are not only carcinogenic but also mutagenic and teratogenic, meaning they can cause severe disruptions in cellular function, resulting in a broad spectrum of biological damage (Zhang et al., 2022; Parmar et al., 2024).

One of the primary environmental concerns regarding PAHs is their accumulation in natural ecosystems, particularly in forested areas, which act as “nature’s sinks” for pollutants. These areas serve as repositories for atmospheric PAHs that settle through wet and dry deposition (Gong et al., 2021). Once in the soil, PAHs can persist for decades, altering microbial communities, affecting plant growth, and moving through the food web. Forests, especially in industrial regions or near urban centers, are often subjected to high levels of PAH deposition, leading to a pressing need for sustainable and effective remediation strategies (Sakshi et al., 2019; Gong et al., 2021; Parmar et al., 2024).

Phenanthrene, pyrene, naphthalene, and fluoranthene are key PAHs of concern in this study due to their widespread occurrence, persistence in the environment, and toxicities (Sakshi et al., 2019; Parmar et al., 2024). These compounds represent different molecular structures and ring sizes, which influence their bioavailability, degradation rates, and interactions with living organisms (Sakshi et al., 2019; Zhang et al., 2022). Naphthalene, a two-ring PAH, is relatively more volatile and can be acutely toxic to plants and aquatic life, while phenanthrene (three rings) and fluoranthene (four rings) can exhibit longer environmental persistence and greater potential for bioaccumulation (Sakshi et al., 2019). Pyrene, another four-ring PAH, is particularly significant because of its recalcitrance to degradation and its ability to induce oxidative stress in plants (Houshani et al., 2019). Studying these PAHs collectively allows researchers to understand a spectrum of plant and microbial responses, contributing to the development of more comprehensive remediation strategies (Sakshi et al., 2019; Parmar et al., 2024).

Phytoremediation, the use of plant holobionts to clean up contaminated environments, has garnered increasing attention as a cost-effective, eco-friendly, and sustainable long-term option for remediating PAH-contaminated soils. This technique leverages the natural processes of plant metabolism, root exudation, and microbial symbiosis to degrade, stabilize, or extract harmful pollutants from the soil. Phytoremediation offers numerous advantages over conventional chemical or mechanical remediation methods, which are often costly, disruptive to ecosystems, and limited in their long-term effectiveness (Shi et al., 2022).

Within the context of phytoremediation, different plant species have varying abilities to tolerate, accumulate, or degrade PAHs, depending on their root systems, metabolic capacities, and interactions with soil microorganisms (Alkio et al., 2005; Zabłudowska et al., 2009; Afegbua and Batty, 2018). Among these, the black alder (*Alnus glutinosa* (L.) Gaertn.) has emerged as a promising candidate for remediation due to its unique biological and ecological characteristics and proven remediation capacity for other pollutants (cadmium (Nahvi et al., 2024), α, β, and δ hexachlorocyclohexane (Amirbekov et al., 2024) and δ hexachlorocyclohexane (Košková et al., 2022). Black alder trees, native to Europe and parts of Asia, are highly adaptable and resilient, often thriving in nitrogen-poor soils and wet environments where other trees might struggle (Košková et al., 2022). Alder ability to fix atmospheric nitrogen through symbiosis with the actinobacteria *Frankia* can also likely support their growth in stressed or contaminated environments (Deptuła et al., 2020).

While black alder trees likely possess inherent capabilities to potentially tolerate and manage PAH-contaminated environments as they do with other pollutants (Košková et al., 2022; Amirbekov et al., 2024; Nahvi et al., 2024), their phytoremediation potential could likely be significantly enhanced through strategic plant-microbe interactions (Tervahauta et al., 2009). The addition of microorganisms, either native or foreign, to contaminated sites or soils, where they work to break down the pollutants is called bioaugmentation (Sakshi et al., 2019; Parmar et al., 2024). Specific microbial strains have evolved the ability to degrade PAHs, transforming them into less harmful compounds through enzymatic pathways (Cunliffe and Kertesz, 2006; Sakshi et al., 2019). Moreover, other plant growth promoting microorganisms could be used as well. Hence by fostering symbiotic relationships with these microorganisms, the capacity to remediate PAH-contaminated soils could be further boosted. This optimization can work both ways. Either the trees’ or the microorganisms’ (or both) ability to degrade PAH could be amplified in both direct and indirect ways (Germaine et al., 2009; Rezek et al., 2009; Shi et al., 2022).

Potential microbial allies in this context include bacterial strains such *as Pseudomonas putida* Trevisan and *Sphingobium yanoikuyae* Yabuuchi et al. These microorganisms possess specific enzymatic systems capable of breaking down PAHs into simpler compounds that are more easily metabolized by plants or further degraded by soil microbes (Cunliffe and Kertesz, 2006; Wang et al., 2021; Cui et al., 2024). Additionally, our preliminary tests revealed that yeast strain like *Rhodotorula* sp*haerocarpa* (S.Y. Newell & Fell) Q.M. Wang, F.Y. Bai, M. Groenewald & Boekhout may also be a beneficial black alder symbiont promoting growth and/or resilience (unpublished results). The selection of microbial strains should be tailored to the specific conditions, as different microbes may have varying affinities for different PAH compounds, different plant populations, etc (Mastretta et al., 2009; Pawlik et al., 2017).

One area of emerging interest is genetic variability among plant populations and its influence on their remediation efficiency (Thygesen and Trapp, 2002; Tervahauta et al., 2009; Yang et al., 2015; Iori et al., 2017; Salam et al., 2022). Different genetic groups of black alder may exhibit varying tolerance to PAH stress, growth patterns, and interactions with microbial communities. Certain genotypes might be more effective at recruiting PAH-degrading microbes or exhibit enhanced metabolic pathways for dealing with oxidative stress caused by PAHs (Tervahauta et al., 2009; Salam et al., 2022). Testing different genetic lines of black alders could therefore provide valuable insights into optimizing their use in remediation projects.

To effectively evaluate the impact of PAHs on black alder and its microbial partners, it is essential to assess various physiological and biochemical parameters. Tree growth metrics provide insights into the overall health and stress tolerance of the tree (Pietrini et al., 2009; Iori et al., 2017; Salam et al., 2022). In addition, monitoring primary and secondary metabolism in black alders can reveal how the tree is metabolically responding to PAH stress (Pietrini et al., 2009; Iori et al., 2017; Molina and Segura, 2021; Xia et al., 2021). Furthermore, the antioxidant response system of black alders plays a crucial role in mitigating the oxidative stress induced by PAHs. PAH exposure can lead to the generation of reactive oxygen species (ROS) in plant tissues, which, if unchecked, can cause cellular damage. The activation of antioxidant enzymes then helps mitigate ROS damage and supports the plant’s resilience under pollutant stress (Liu et al., 2009).

Thus, the aim of this study was to evaluate black alder genetic groups, i.e., half-sib families, and their potential microbial partners (selected strains of *Pseudomonas putida*, *Sphingobium yanoikuyae*, and *Rhodotorula* sp*haerocarpa*) in terms of alder stress response to four PAHs (pyrene, naphthalene, fluoranthene, phenanthrene) within a hydroponic system (to limit any variables) in hopes of optimizing the bioremediation process of PAH-contaminated substrates using a black alder-microorganism complex.

## 2 Materials and methods

Black alder seeds were collected from trees growing in the seed plantation of the Šiauliai City Forest Department in 2021. Based on a previous experiment (Striganavičiūtė et al., 2024a), two half-sib families (sharing one known parent, with the other unknown), 13-99-1K and 42-65-7K, selected for their resistance to polycyclic aromatic hydrocarbons (PAHs), were used in the current 2023–2024 study. The seedlings were inoculated with PAH-degrading bacteria—*Pseudomonas putida* (*P.p.*), *Sphingobium yanoikuyae* (*S.y.*), and yeast *Rhodotorula* sp*haerocarpa* (*R.s.*) (Cunliffe and Kertesz, 2006; Wang et al., 2021; Cui et al., 2024) —and exposed to four PAHs—phenanthrene (PHE), pyrene (PYR), naphthalene (NAPH), and fluoranthene (FLUO)—at a concentration of 2 mg L−¹. Growth parameters, secondary metabolites, photosynthetic pigments, and antioxidant enzyme activities were evaluated. Control groups were exposed only to pollutants, with untreated plants (no pollutant or microorganism) also shown in the graphs. The experiment included 40 groups, each with 20 plants. Biochemical analyses were performed on three biological replicates per treatment, with each replicate consisting of leaves pooled from at least five different plants. The picture of the trial (**Supplementary Figure S1**) is shown in the **Supplementary Materials**.

### 2.1 Microorganisms

The microorganisms used—*Pseudomonas putida* Trevisan (*P.p.*), DSM No. 28064, *Sphingobium yanoikuyae* Yabuuchi et al. (*S.y.*), DSM No. 6900 (both obtained from the Leibniz Institute DSMZ) —were selected for their ability to degrade PAHs, colonize plant tissues, and be non-toxic to humans. Yeast *Rhodotorula* sp*haerocarpa* (S.Y. Newell & Fell) Q.M. Wang, F.Y. Bai, M. Groenewald & Boekhout (*R.s.*), MUCL No. 30605 (sourced from the Belgian Coordinated Collections of Microorganisms, BCCM) was picked for its previously observed beneficial impact on alder growth and/or resilience (unpublished results from a pre-trial). The strains were cultured on solid low-salt Lysogeny Broth (LB) medium (Duchefa Biochemie, Haarlem, Netherlands) at approximately 25°C, then transferred to liquid LB medium (pH 7) and incubated for 3 days at 30°C before inoculation.

### 2.2 Seed inoculation and plant setup for experimental procedures

To inoculate black alder seeds, 120 mL of microorganism cultures were centrifuged at 3500 × g for 5 minutes to pellet the cells, which were then washed three times with 0.9% NaCl solution. The optical density of each microorganism solution was adjusted to 1 at 600 nm.

Five hundred seeds per half-sib family were soaked in 20 mL of the microorganism solution for 30 minutes, while control groups were soaked in 0.9% NaCl solution. After soaking, the seeds were air-dried. The seeds were not sterilized prior to the experiment, as the natural microorganisms on their surface are essential for plant growth. Microbial contaminants were not excluded, ensuring that all experimental groups were treated equally.

The seeds were then sown in hydroponic rockwool cubes, which had been soaked in distilled water (pH 5.6) for 24 hours. The cubes were irrigated with a full-strength (100%) Hoagland nutrient solution (pH 5.6). The composition of the modified nutrient solution included 6 mM KNO_3_, 2.32 mM Ca(NO_3_)_2_ × 4 H_2_O, 1.86 mM MgSO_4_ × 7 H_2_O, 1 mM NH_4_H_2_PO_4_, 46 μM H_3_BO_3_, 9 μM MnCl_2_ × 4H_2_O, 8.99 μM C_12_H_12_Fe_2_O_18_, 0.76 μM ZnSO_4_ × 7 H_2_O, 0.5 μM CuSO_4_ × 5 H_2_O, and 0.58 μM Na_2_MoO_4_ × 2 H_2_O.

Seeds were kept in darkness for one week at 25/20°C, after which they were exposed to white light (94.5 μmol m^−2^ s^−1^) for an additional four weeks, until reaching the four-true-leaf stage (growth stage BBCH14, as described by Guilayn et al. (2020). During this period, the plants were watered with a half-strength (50%) Hoagland nutrient solution.

### 2.3 Hydroponic setup, experimental conditions, and usage of PAHs

Black plastic containers (10 L, 30 × 25 × 12 cm) were insulated with 2 cm thick polystyrene foam boards. A custom plastic netting panel with 20 holes was used to hold hydroponic rockwool cubes containing seedlings with four true leaves.

Aeration was provided by air pumps (Union Star Air AC-500, 230 V, 50 Hz, 2 W, China) connected to plastic hoses with non-return valves (12 × 7 × 3 cm), along with 2 cm air pebbles. The seedlings were grown for four in a growth chamber at 25/20°C (day/night) with a 16/8 h photoperiod of white light, providing an irradiance of 94.5 μmol m^−2^ s^−2^.

The containers were filled with 7 L of full-strength Hoagland’s nutrient solution, to which 2 mg L−¹ of each pollutant (phenanthrene, pyrene, naphthalene, and fluoranthene—all purchased from Acros Organics, Belgium) dissolved in acetone was added. A control group with acetone only showed no significant effect.

At the end of the period, shoot and root lengths were recorded. The mean shoot length was calculated both at the start and conclusion of the experiment to evaluate growth, while the longest root length was measured only as the maximum distance from the root tip to the root base for each seedling at the end of the experiment. For biochemical analyses, leaves were harvested, and six replicates of 0.1 g were weighed for each experimental group (three for photosynthetic pigment analysis and three for antioxidant enzyme tests).

### 2.4 Biochemical assessment

#### 2.4.1 Sample preparations for antioxidant enzyme activity assessment

The extraction of samples was conducted according to the methods described in Čėsnienė et al. (2023). In brief, fresh biomass (3 × 0.1 g per group) was performed using liquid nitrogen and subsequently mixed with an extraction buffer containing K-phosphate buffer (0.25 M pH 7.8), Triton-X, polyvinylpolypyrrolidone, and ascorbic acid (ASC). After centrifugation at 21,910 × g and -4°C for 1 hour, the supernatant was used for enzyme assays (catalase (CAT), superoxide dismutase (SOD), ascorbate peroxidase (APX), guaiacol peroxidase (POX), glutathione reductase (GR), and glutathione S-transferase (GST)) and protein quantification. For further purification of the enzymes APX, POX, GR, and GST, the supernatant was passed through Sephadex G-25 columns (Column PD-10, Cytiva, Gillingham, UK) (Kozlowski et al., 1999)., and the filtered bulk (non-fractionated) extract was used for subsequent analyses. All extraction procedures were conducted on ice to maintain sample integrity (Singleton et al., 1999).

#### 2.4.2 Total protein determination

The concentration of total protein (PROT) was assessed using the methodology outlined in Čėsnienė et al. (2023), which details the procedures and formulas used. In summary, PROT concentrations in the crude extract were quantified using the Bradford assay (Bradford, 1976), based on the reaction of peptide bonds with Cu^2+^ ions. Absorbance was measured at 660 nm and expressed as micrograms of Bovine Serum Albumin equivalent per milliliter of crude extract.

#### 2.4.3 Catalase enzyme activity determination

CAT activity was measured using the method and calculations described in Čėsnienė et al. (2023), which involves reacting the extract with H_2_O_2_ (Kar and Mishra, 1976) and K-phosphate buffer (0.25 M pH 7). Absorbance was recorded at a wavelength of 240 nm at regular intervals, and activity was calculated.

#### 2.4.4 Superoxide dismutase enzyme activity determination

SOD activity was assessed using the method and calculations described in Čėsnienė et al. (2023), by mixing the extract with a reaction buffer (K-phosphate buffer (0.1 M pH 7.8), methionine, nitro blue tetrazolium, ethylenediamine tetraacetic acid (EDTA), and riboflavin). The mixture was exposed to white light (irradiance 30 μmol m−² s−²) until the samples became darker than the control, and absorbance was at 550 nm to calculate activity, following the procedure in the cited publication.

#### 2.4.5 Guaiacol peroxidase enzyme activity determination

POX activity was assessed using the method described in Čėsnienė et al. (2023), by combining the filtered extract (Kar and Mishra, 1976; Wu and von Tiedemann, 2002) with pyrogallol in a K-phosphate buffer (0.25 M pH 6.5) and 10% H_2_O_2_. Absorbance was recorded at 430 nm, monitoring changes over time at 35-second intervals.

#### 2.4.6 Ascorbate peroxidase enzyme activity determination

The method outlined in Čėsnienė et al. (2023) was utilized for APX assays, where the filtered extract (Nakano and Asada, 1981; Cakmak and Marschner, 1992; Wu and von Tiedemann, 2002) was mixed with ASC solution and a 10% H_2_O_2_ solution. APX activity was monitored at 290 nm by measuring the decrease in absorbance over time at intervals of 35 seconds.

#### 2.4.7 Glutathione S-transferase enzyme activity determination

GST activity follows the procedure described in the Striganavičiūtė et al. (2024a) publication. It was measured by combining the extract with reaction buffer and 10 mM GSH. The reaction buffer contained 1-chloro-2,4-dinitrobenzene (Thermo Fisher, Germany) in K-phosphate buffer (0.25 M pH 6.5), and the GSH solution was prepared in K-phosphate buffer (pH 7.4). Absorbance at 340 nm was recorded every 40 seconds for six intervals.

#### 2.4.8 Glutathione reductase enzyme activity determination

To assess glutathione reductase (GR) enzyme activity, the extract was combined with a reaction buffer containing HEPES buffer (pH 8), EDTA, and NADPH. Following the addition of GSSG, changes in absorbance at 340 nm were monitored over time. Detailed procedures for preparation and calculations are provided in Čėsnienė et al. (2023).

#### 2.4.9 Extraction preparation for analyzing photosynthetic pigments, secondary metabolites, malondialdehyde, and sugar levels

Fresh leaves (3 × 0.1 g per group) ground using a “Precellys 24” tissue homogenizer (Bertin Technologies, France) at 1956 × g for 30 seconds. 80% ethanol (v/v) was added, and the mixture was homogenized again 30 seconds. The samples were then centrifuged at 21,910 × g for 30 minutes at 4°C using a Hettich Universal 32R centrifuge (Andreas Hettich GmbH and Co. KG, Germany), and the supernatant was collected for analysis of chlorophyll, secondary metabolites, malondialdehyde (MDA), and soluble sugars.

#### 2.4.10 Determination of total phenolic content

TPC was measured using a modified Lowry method involving Folin-Ciocalteu reagent. The extract was mixed with the reagent (1:9, w/v), incubated for 5 minutes, and sodium carbonate was added. The solution was kept in the dark 1 hour before the absorbance was recorded at 725 nm with a microplate reader. For the complete protocol, refer to the work of Čėsnienė et al. (2023).

#### 2.4.11 Determination of total flavonoid content

TFC was determined by forming a flavonoid-Al (III) complex, as described by Chang et al. (2020). Extracts were combined with a reaction buffer containing absolute ethyl alcohol, aluminum chloride solution, potassium acetate, and distilled water in 96-well microplates. Absorbance was measured at 415 nm to quantify TFC. Detailed procedures and calculations can be found in the methodology section of Čėsnienė et al. (2023).

#### 2.4.12 Determination of chlorophyll a and b (CHL), and total carotenoids

The absorption levels of the extract (supernatant) were recorded at wavelengths of 470 nm, 648 nm, and 664 nm using a SpectroStar Nano microplate reader (BMG Labtech, Offenburg, Germany) in 96-well microplates. The analysis employed specific formulas and models as outlined in Čėsnienė et al. (2023). The ratio of chlorophyll *a* to chlorophyll *b* was calculated by dividing the absorbance value of chlorophyll *a* by that of chlorophyll *b*.

#### 2.4.13 Determination of malondialdehyde

For the analysis of MDA, the supernatant was combined with a reaction mixture consisting of trichloroacetic acid (Molar Chemicals Kft, Hungary) and thiobarbituric acid (Alfa Aesar, Germany), following the procedure outlined by Čėsnienė et al. (2023). The mixture was incubated at 95°C for 30 minutes, then cooled on ice. The absorbance of the supernatant was measured at wavelengths of 440 nm, 532 nm, and 600 nm.

#### 2.4.14 Determination of soluble sugars

The determination of SS was performed by mixing the sample with anthrone reagent (Carl Roth, Germany). The anthrone reagent was prepared by dissolving 0.1 g of anthrone in 100 mL of concentrated H_2_SO_4_ (Chempur, Poland), following the method described by Čėsnienė et al. (2023). The mixture was incubated at 90°C for 1 hour, after which the absorbance was measured at 620 nm.

### 2.5 Statistical analysis

Statistical analyses were performed using SPSS (version 28.0.1.1 IBM Inc.) and R software (version 4.3.1). SPSS was used for preliminary comparisons to assess whether specific treatment groups significantly differed from control groups. The Kruskal-Wallis H test for independent samples as a non-parametric alternative to one-way ANOVA. Further pairwise comparison on ranks was done with *Dunn’s* test (*p<0.05*). The confidence levels were 95%. Pairwise comparisons from *Dunn’s* test were reported with adjusted (Bonferroni correction) *p*-values to indicate which specific groups differed significantly.

To investigate the effects of Family, Pollutant, and Microorganism on various parameters, including Shoot, Root, MDA, CAT, and GST, a three-way Analysis of Variance (ANOVA) was performed in R. This statistical technique evaluated the main effects of these factors and their interactions. The analysis was performed on a dataset containing columns for Family, Pollutant (including untreated plants to better observe the effect of pollutants on black alder seedlings), Microorganism, and several response variables. Missing values were addressed by omitting rows with incomplete data to ensure the reliability of the ANOVA results.

Before conducting the ANOVA, the assumption of homogeneity of variance was tested using Levene’s Test, performed with the *leveneTest()* function from the car package in R (Fox and Weisberg, 2011). For parameters where Levene’s Test was non-significant (i.e., Shoot, Root, MDA, CAT, and GST), the variances across groups were homogeneous, meeting the assumption required for ANOVA. A three-way ANOVA was executed using the *aov()* function in R (R Core Team, 2021) to evaluate the main effects and interactions of Family, Pollutant, and Microorganism.

For parameters where Levene’s Test was significant (i.e., CHL, TPC, TFC, APX, POX, GR, SOD, CAR, and SS), indicating variance heterogeneity, Welch ANOVA was performed using the *oneway.test()* function in R. Pairwise comparisons were conducted using the Wilcoxon rank-sum test with continuity correction to determine specific group differences, with results adjusted for multiple comparisons using the Bonferroni method.

*Post hoc* analyses were conducted using the *emmeans* package for parameters analyzed with the three-way ANOVA. Pairwise comparisons were performed to identify significant differences between group levels, and the results were filtered to highlight comparisons with p-values less than 0.05, indicating statistical significance.

To investigate and visualize the multivariate response patterns across all variables, principal component analysis (PCA) was conducted to compare the influence of Family, Pollutant, and Microorganism on the physiological parameters. The PCA was performed using the *prcomp()* function in R, based on the standardized values of the measured parameters. PCA plots were generated with the ggplot2 package, visualizing the first two principal components (PC1 and PC2) and their respective variance explained. Family, Pollutant, and Microorganism were included as grouping factors to explore their effect on the distribution of the samples in the PCA space, with confidence ellipses plotted for each group (95%). Centroids were assigned to each group to represent the average individual.

Statistical analyses in R were primarily used for ANOVA and *post hoc* analyses, and PCA, providing insights into the main effects and interactions of the factors. Welch ANOVA and Wilcoxon test results highlighted specific pairwise differences where necessary, while PCA offered a broader view of the relationship among the groups.

## 3 Results

### 3.1 Morphological responses: shoot growth and root length

**Figure 1:**
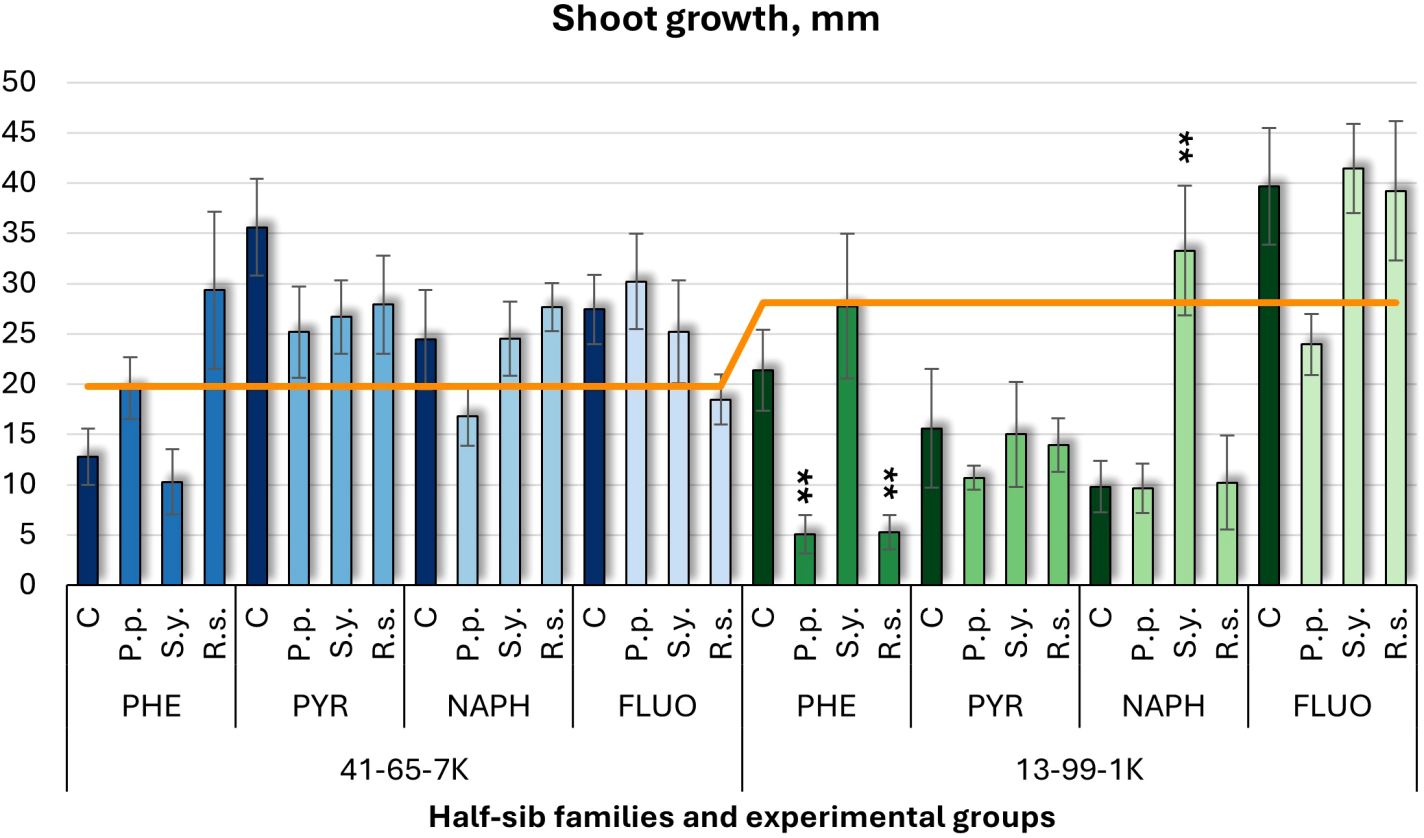
Shoot growth comparison (mm) in black alder half-sib families (41-65-7K and 13-99-1K) under various microbial inoculations: control (C), *Pseudomonas putida* (*P.p*.), *Sphingobium yanoikuyae* (*S.y.*), and *Rhodotorula* sp*haerocarpa* (*R.s.*), across different pollutant treatments: phenanthrene (PHE), pyrene (PYR), naphthalene (NAPH), and fluoranthene (FLUO). The orange line indicates the mean shoot growth in the absence of pollutants and microbial inoculations. Each pollutant treatment includes a specific control (C), representing plants treated with that specific pollutant but without microbial inoculation by which statistical significance was assessed. Each treatment consisted of 20 plants. Data are presented as mean ± standard error (SE). Statistically significant differences from the control group were assessed using the Kruskal-Wallis H test: **p<0.01.

**Figure 2:**
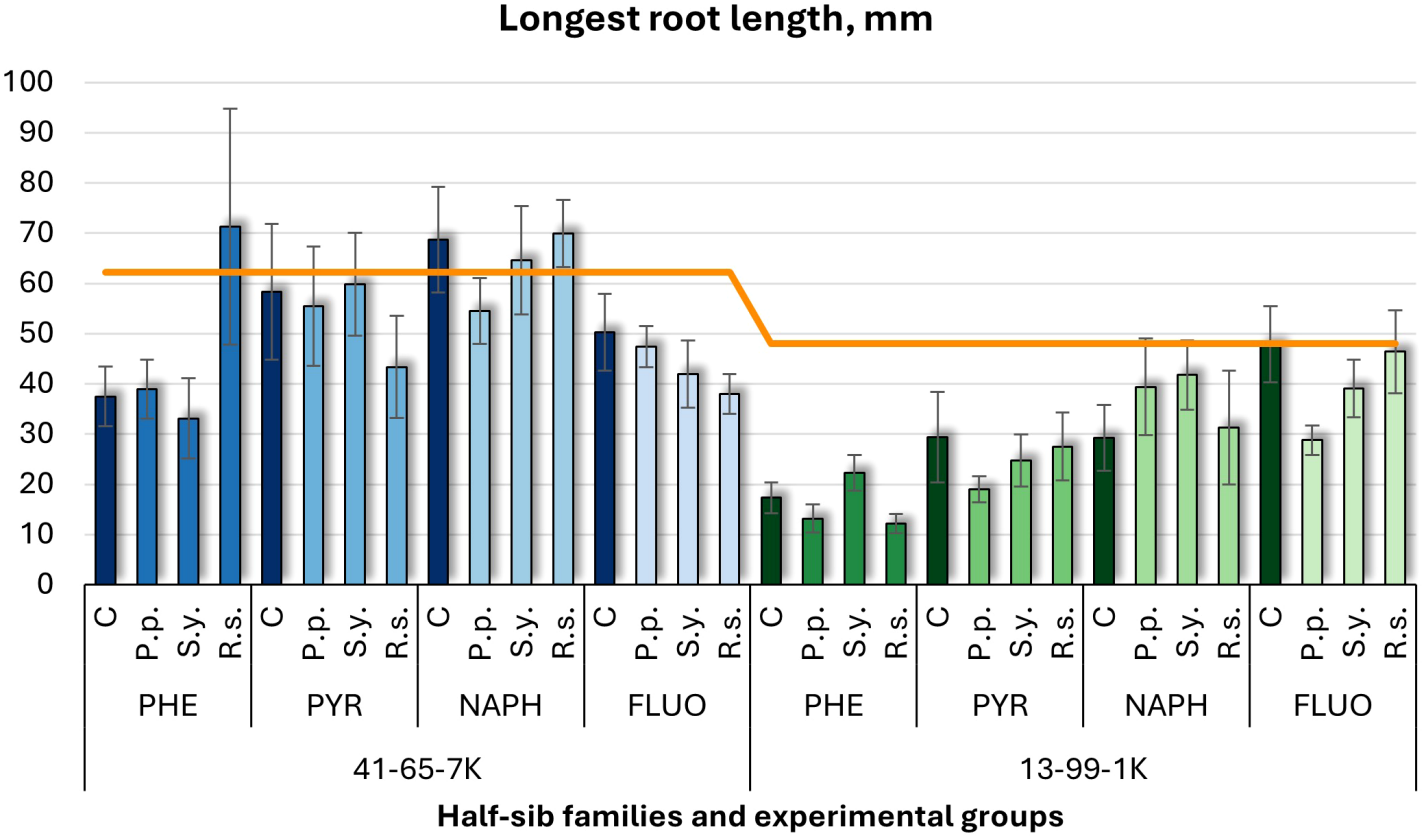
Longest root length comparison (mm) in black alder half-sib families (41-65-7K and 13-99-1K) under various microbial inoculations: control (C), *Pseudomonas putida* (*P.p*.), *Sphingobium yanoikuyae* (*S.y.*), and *Rhodotorula* sp*haerocarpa* (*R.s.*), across different pollutant treatments: phenanthrene (PHE), pyrene (PYR), naphthalene (NAPH), and fluoranthene (FLUO). Each pollutant treatment includes a specific control (C), representing plants treated with that specific pollutant but without microbial inoculation. Each treatment consisted of 20 plants. The orange line indicates the mean root length in the absence of pollutants and microbial inoculations. Data are presented as mean ± standard error (SE).

Shoot growth (**Figure 1**) and longest root length (**Figure 2**) were measured. In the half-sib family 13-99-1K seedlings, *Pseudomonas putida* (*P.p.*) and *Rhodotorula* sp*haerocarpa* (*R.s.*) inoculations reduced shoot growth up to four times under phenanthrene (PHE) treatment, while *Sphingobium yanoikuyae* (*S.y.*) increased it more than three times under naphthalene (NAPH). Control groups exposed to pollutants showed lower shoot growth than the untreated control (orange line), except under fluoranthene (FLUO) treatment. In the seedlings of half-sib family 41-65-7K, microbial treatments had minimal effects, and pollutant-exposed controls generally had higher shoot growth than the untreated group, except under PHE.

A three-way ANOVA (**Supplementary Table S2**) showed a significant effect of Pollutant (*p* < 0.001) on shoot growth, with Tukey *post hoc* analysis (**Supplementary Table S13**) confirming differences between NAPH and FLUO, FLUO and PHE, and PYR and NAPH.

For longest root length (**Figure 2**), PHE led to shorter root lengths in both families. In 41-65-7K, pyrene (PYR) and NAPH resulted in similar root lengths to the untreated group, while FLUO led to slightly shorter roots. In the 13-99-1K seedlings, *P.p.* and *S.y.* caused shorter root lengths, particularly under PHE treatment, with most experimental groups below untreated control, except for the control and *R.s.* under FLUO treatment.

Three-way ANOVA (**Supplementary Table S3**) indicated significant effects of Family (*p* < 0.001) and Pollutant (*p* < 0.001), with an interaction between Family and Pollutant approached significance (*p* = 0.0721). *Post hoc* analysis (**Supplementary Table S8**) confirmed differences between families, and Tukey *post hoc* tests (**Supplementary Table S13**) revealed significant contrasts between the control and PHE, NAPH, and FLUO treatments.

### 3.2 Oxidative stress response: malondialdehyde levels

**Figure 3:**
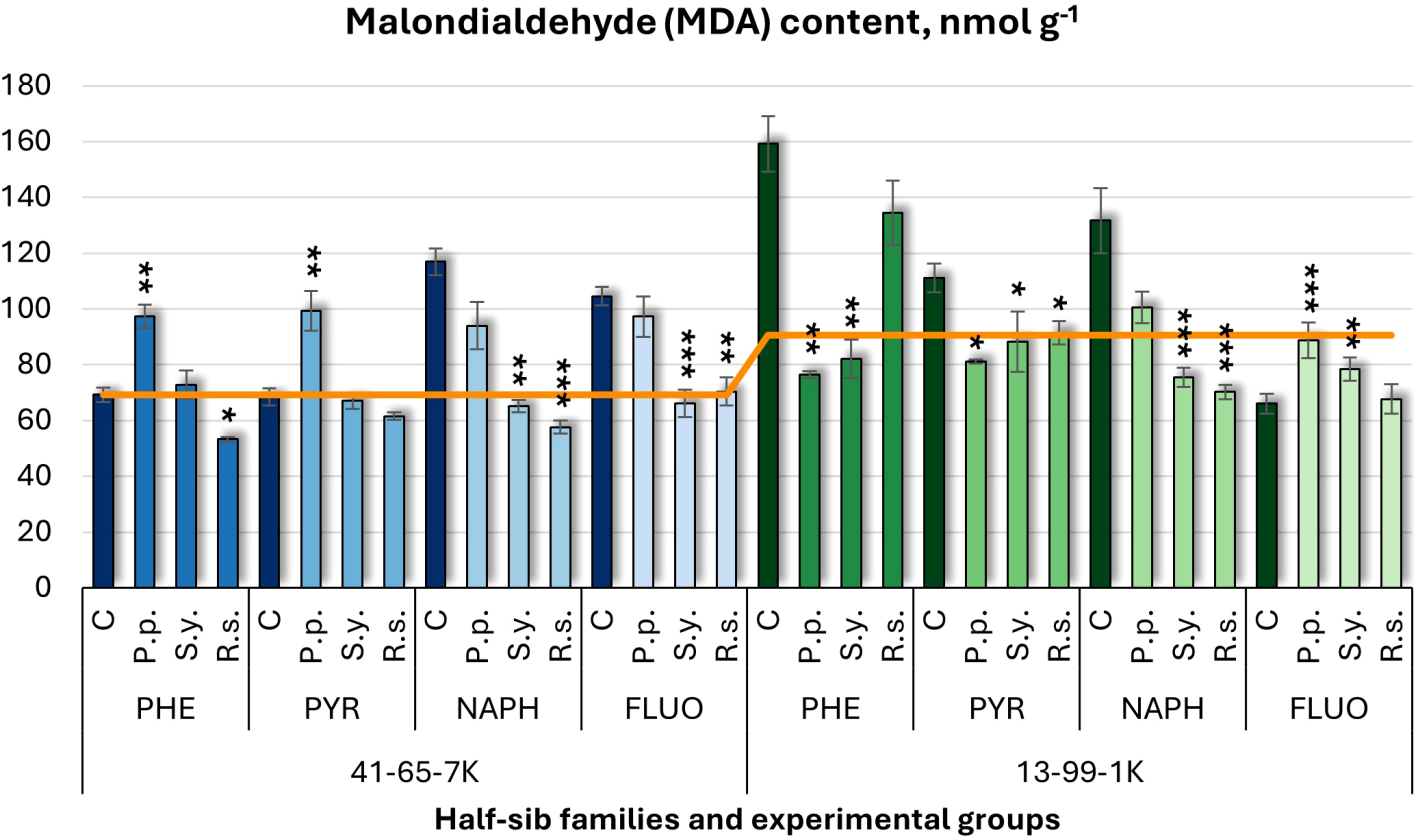
Malondialdehyde (MDA) content (nmol g^-1^ fresh weight) comparison in black alder half-sib families (41-65-7K and 13-99-1K) under various microbial inoculations: control (C), *Pseudomonas putida* (*P.p*.), *Sphingobium yanoikuyae* (*S.y.*), and *Rhodotorula* sp*haerocarpa* (*R.s.*), across different pollutant treatments: phenanthrene (PHE), pyrene (PYR), naphthalene (NAPH), and fluoranthene (FLUO). The orange line indicates the mean MDA content in the absence of pollutants and microbial inoculations. Each pollutant treatment includes a specific control (C), representing plants treated with that specific pollutant but without microbial inoculation by which statistical significance was assessed. Biochemical analyses were conducted on three biological replicates per treatment, with each replicate consisting of leaves pooled from at least five different plants. Data are presented as mean ± standard error (SE). Statistically significant differences from the control group were assessed using the Kruskal-Wallis H test: *p<0.05; **p<0.01; ***p<0.001.

Malondialdehyde (MDA) levels (**Figure 3**), varied with microbial inoculations. In the half-sib family 41-65-7K, *P.p.* increased MDA levels under PHE and PYR treatments. In seedlings of half-sib family 13-99-1K, *P.p.* reduced MDA levels by 52% in PHE and 27% in PYR but increased it in FLUO. *S.y.* decreased MDA levels in the 41-65-7K seedlings under NAPH and FLUO, while in the 13-99-1K family, *S.y.* reduced MDA levels under PHE, PYR, and NAPH treatments but increased them under FLUO. *R.s.* decreased MDA levels in the 41-65-7K seedlings by 50% under NAPH, and also reduced it in PHE and FLUO, while 13-99-1K family, *R.s.* reduced MDA by 46% under NAPH and reduced it under PYR. Compared to untreated plants, MDA levels in control groups were higher in the 41-65-7K seedlings under NAPH and FLUO, while in the 13-99-1K family, they increased under PHE, PYR, and NAPH treatments, but decreased under FLUO treatment.

Three-way ANOVA (**Supplementary Table S4**) showed significant effects of Family, Pollutant, and Microorganism (*p* < 0.001), with all two-way and three-way interactions also significant (*p* < 0.001). *Post hoc* analyses (**Supplementary Tables S8**, **S10**) revealed significant differences in MDA between microorganisms and control, as well as between *P.p.* and *R.s.*, and *P.p.* and *S.y.*, but not *R.s.* and *S.y.* Tukey *post hoc* tests (**Supplementary Table S13**) revealed significant variations between the control and PHE, as well as among FLUO and NAPH, PHE and NAPH, PYR and NAPH, and PYR and PHE.

### 3.3 Photosynthetic pigment responses: chlorophyll *a/b* ratio and carotenoid concentration

**Figure 4:**
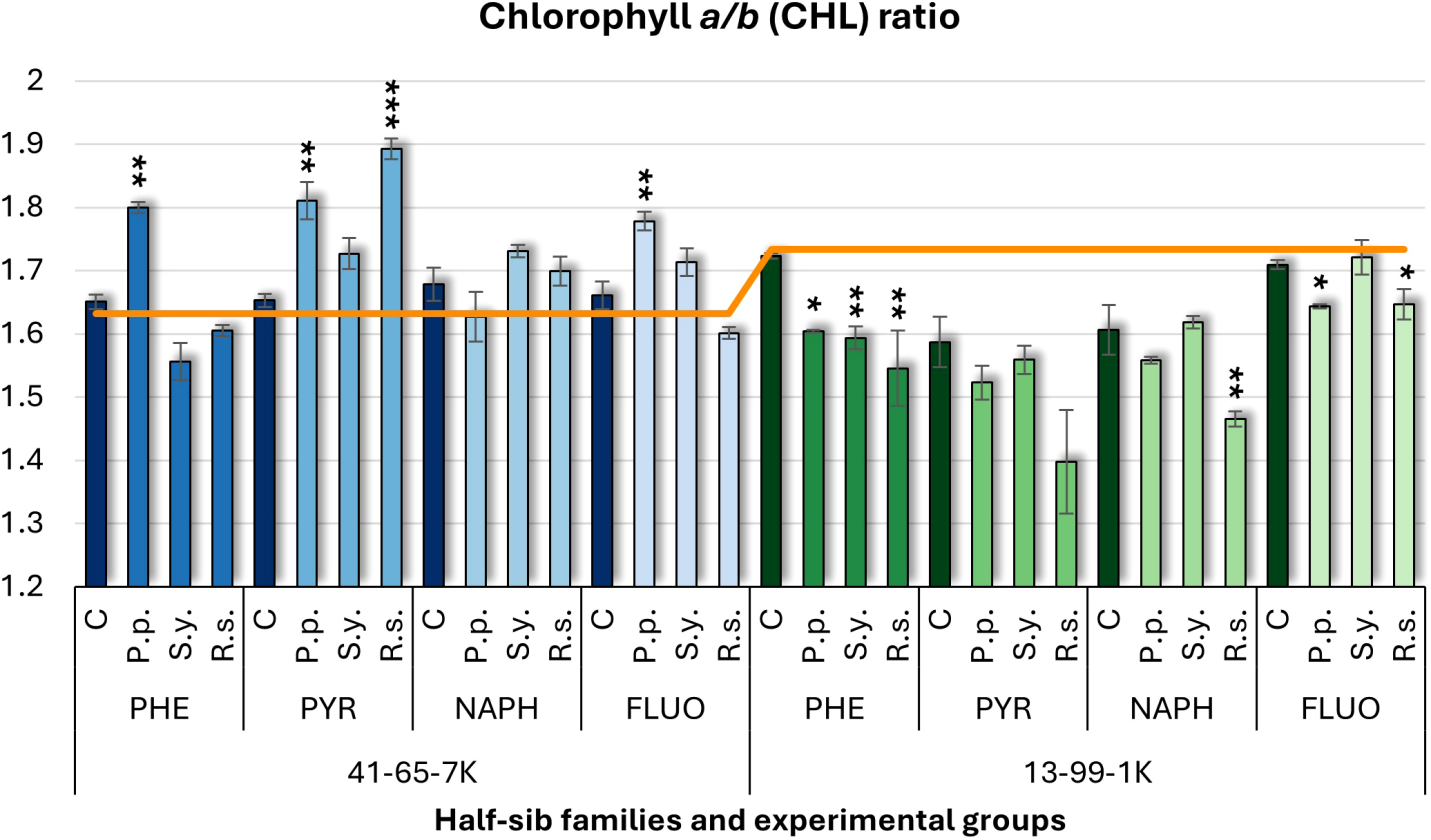
Chlorophyll *a/b* (CHL) ratio comparison in black alder half-sib families (41-65-7K and 13-99-1K) under various microbial inoculations: control (C), *Pseudomonas putida* (*P.p*.), *Sphingobium yanoikuyae* (*S.y.*), and *Rhodotorula* sp*haerocarpa* (*R.s.*), across different pollutant treatments: phenanthrene (PHE), pyrene (PYR), naphthalene (NAPH), and fluoranthene (FLUO). The orange line indicates the mean CHL ratio in the absence of pollutants and microbial inoculations. Each pollutant treatment includes a specific control (C), representing plants treated with that specific pollutant but without microbial inoculation by which statistical significance was assessed. Biochemical analyses were conducted on three biological replicates per treatment, with each replicate consisting of leaves pooled from at least five different plants. Data are presented as mean ± standard error (SE). Statistically significant differences from the control group were assessed using the Kruskal-Wallis H test: *p<0.05; **p<0.01; ***p<0.001.

For the chlorophyll *a/b* (CHL) ratio (**Figure 4**), contrasting effects were observed between the two half-sib families. In the 41-65-7K family, *P.p.* inoculation increased the CHL ratio under PHE, PYR, and FLUO, whereas in the 13-99-1K family, *P.p.* decreased it under PHE and FLUO treatments. *S.y.* consistently reduced the CHL ratio in the 13-99-1K seedlings when exposed to PHE. *R.s*. inoculation led to an increased CHL ratio in the 41-65-7K seedlings under PYR treatment but decreased it in the 13-99-1K seedlings when exposed to PHE and FLUO. Compared to the untreated control, the 13-99-1K seedlings had lower CHL ratios under PYR and NAPH treatments, while all other groups had CHL ratios similar to the untreated plants.

Welch ANOVA (**Supplementary Table S7**) indicated significant Family × Pollutant × Microorganism interaction for CHL (*p* < 0.001). Pairwise Wilcoxon test results (**Supplementary Table S9**) revealed significant Family differences, but no significant differences for Microorganism effects. The pairwise Wilcoxon test results (**Supplementary Table S14**) showed significant differences in CHL between control and all pollutant treatments, as well as between NAPH and FLUO.

**Figure 5:**
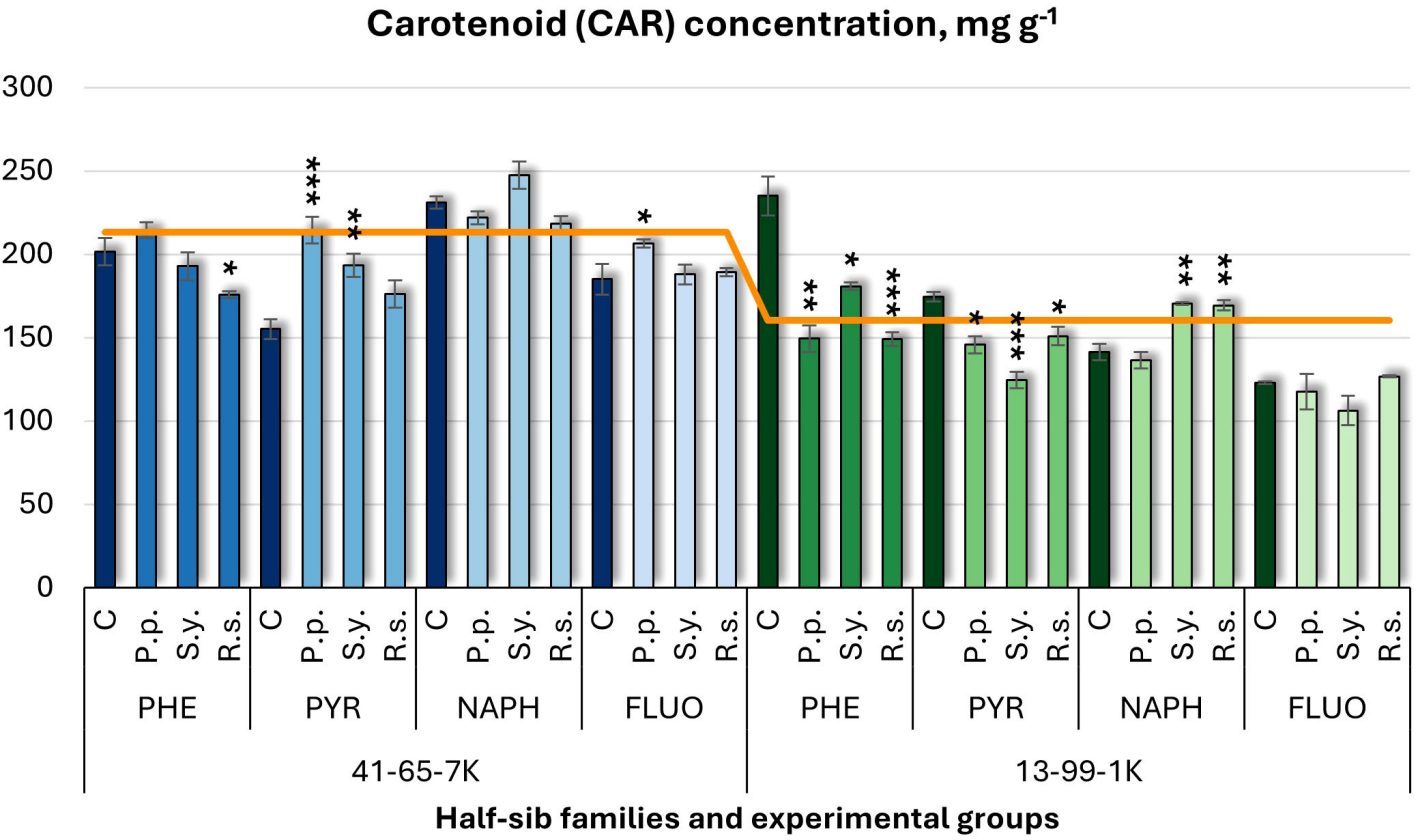
Carotenoid (CAR) concentration (mg g^-1^ fresh weight) comparison in black alder half-sib families (41-65-7K and 13-99-1K) under various microbial inoculations: control (C), *Pseudomonas putida* (*P.p*.), *Sphingobium yanoikuyae* (*S.y.*), and *Rhodotorula* sp*haerocarpa* (*R.s.*), across different pollutant treatments: phenanthrene (PHE), pyrene (PYR), naphthalene (NAPH), and fluoranthene (FLUO). The orange line indicates the mean CAR concentration in the absence of pollutants and microbial inoculations. Each pollutant treatment includes a specific control (C), representing plants treated with that specific pollutant but without microbial inoculation by which statistical significance was assessed. Biochemical analyses were conducted on three biological replicates per treatment, with each replicate consisting of leaves pooled from at least five different plants. Data are presented as mean ± standard error (SE). Statistically significant differences from the control group were assessed using the Kruskal-Wallis H test: *p<0.05; **p<0.01; ***p<0.001.

For carotenoid (CAR) concentration (**Figure 5**), the effect of *P.p.* varied by family. In the 41-65-7K seedlings, *P.p.* increased CAR under PYR and FLUO treatments, while in the 13-99-1K family, it reduced CAR by 36% under PHE and also decreased it under PYR treatments. *S.y.* increased CAR in 41-65-7K family under PYR treatment but had variable effects in 13-99-1K seedlings, increasing it under NAPH and decreasing it under PHE and PYR. *R.s.* decreased CAR in the 41-65-7K seedlings under PHE by 37% but increased it in the 13-99-1K seedlings under NAPH. For controls, CAR levels were lower in 41-65-7K half-sib family under PHE but higher in 13-99-1K half-sib family seedlings exposed to pollutants.

Welch ANOVA (**Supplementary Table S7**) showed significant Family × Pollutant × Microorganism interaction for CAR (*p* < 0.001). Pairwise Wilcoxon test (**Supplementary Table S9**), revealed significant Family differences in CAR. *Post hoc* analysis (**Supplementary Table S10**) revealed no significant differences for Microorganism effects. The pairwise Wilcoxon test results (**Supplementary Table S14**) found significant differences in CAR between the control and all pollutant treatments, for example FLUO and NAPH, FLUO and PHE, PYR and NAPH, and PYR and PHE.

### 3.4 Metabolic changes: soluble sugars, total phenolic content, and total flavonoid content

**Figure 6:**
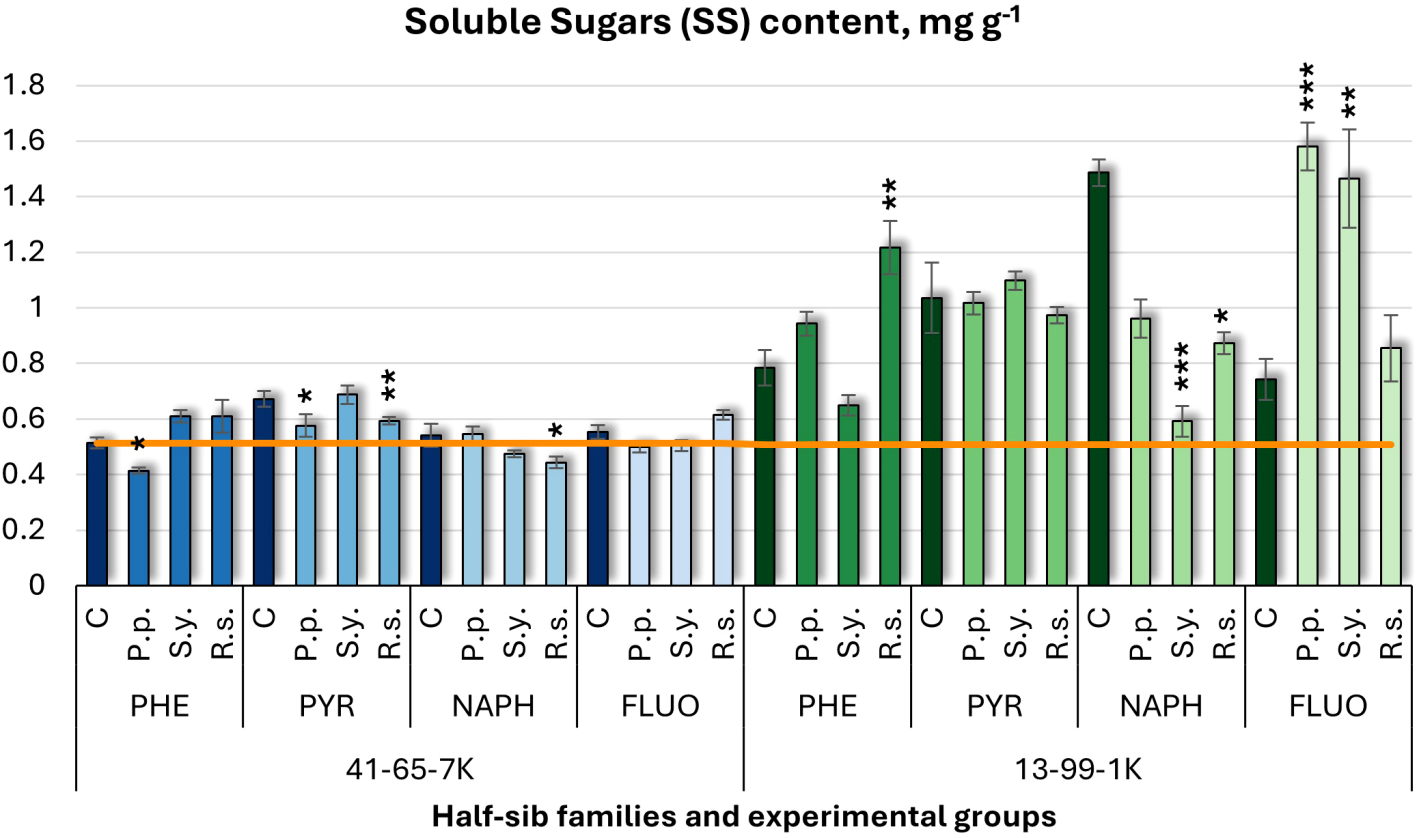
Soluble sugars (SS) content (mg g^-1^ fresh weight) comparison in black alder half-sib families (41-65-7K and 13-99-1K) under various microbial inoculations: control (C), *Pseudomonas putida* (*P.p*.), *Sphingobium yanoikuyae* (*S.y.*), and *Rhodotorula* sp*haerocarpa* (*R.s.*), across different pollutant treatments: phenanthrene (PHE), pyrene (PYR), naphthalene (NAPH), and fluoranthene (FLUO). The orange line indicates the mean SS content in the absence of pollutants and microbial inoculations. Each pollutant treatment includes a specific control (C), representing plants treated with that specific pollutant but without microbial inoculation by which statistical significance was assessed. Biochemical analyses were conducted on three biological replicates per treatment, with each replicate consisting of leaves pooled from at least five different plants. Data are presented as mean ± standard error (SE). Statistically significant differences from the control group were assessed using the Kruskal-Wallis H test: *p<0.05; **p<0.01; ***p<0.001.

For soluble sugars (SS) (**Figure 6**), *P.p.* inoculation decreased SS in the 41-65-7K half-sib family under PHE and PYR but increased it by 2.12-fold in the 13-99-1K half-sib family under FLUO treatment. *S.y.* had variable effects in the 13-99-1K family, increasing SS by 97% under FLUO but decreasing it by 60% under NAPH exposure. *R.s.* inoculation increased SS by 55% in 13-99-1K seedlings under PHE but decreased it under NAPH treatment. In the 41-65-7K family, *R.s.* consistently decreased SS under PYR and NAPH. Overall, the 13-99-1K seedlings had higher SS levels than the untreated control plants (orange line), particularly when grown under PYR and NAPH pollutant conditions.

Welch ANOVA (**Supplementary Table S7**) indicated significant Family × Pollutant × Microorganism interaction (p < 0.001). Pairwise Wilcoxon test (**Supplementary Table S9**) revealed family differences. Significant differences were observed between the control and PYR, and between PYR and both NAPH and PHE. No significant differences for Microorganism effects.

**Figure 7:**
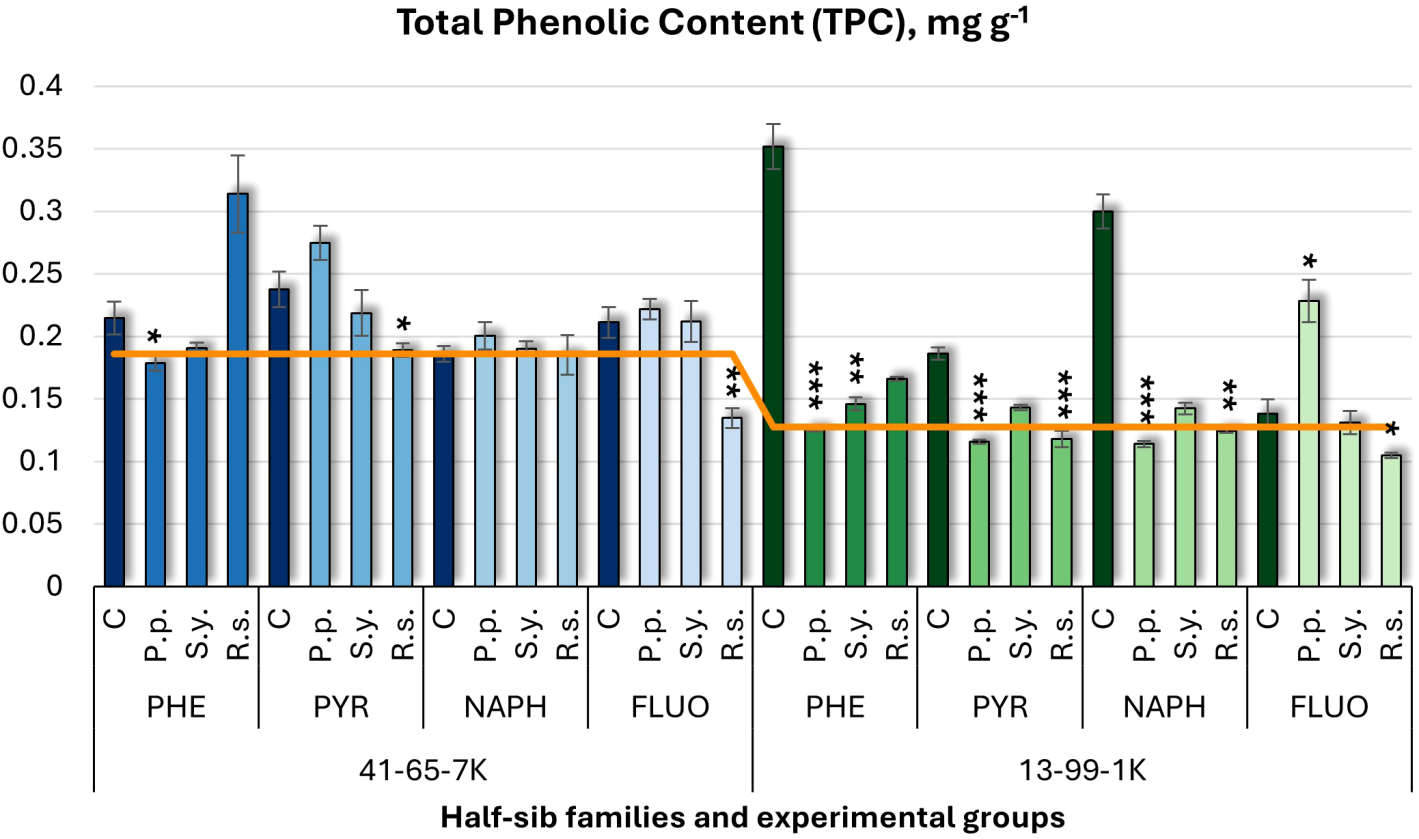
Total phenolic content (TPC) (mg g^-1^ fresh weight) comparison in black alder half-sib families (41-65-7K and 13-99-1K) under various microbial inoculations: control (C), *Pseudomonas putida* (*P.p*.), *Sphingobium yanoikuyae* (*S.y.*), and *Rhodotorula* sp*haerocarpa* (*R.s.*), across different pollutant treatments: phenanthrene (PHE), pyrene (PYR), naphthalene (NAPH), and fluoranthene (FLUO). The orange line indicates the mean of TPC in the absence of pollutants and microbial inoculations. Each pollutant treatment includes a specific control (C), representing plants treated with that specific pollutant but without microbial inoculation by which statistical significance was assessed. Biochemical analyses were conducted on three biological replicates per treatment, with each replicate consisting of leaves pooled from at least five different plants. Data are presented as mean ± standard error (SE). Statistically significant differences from the control group were assessed using the Kruskal-Wallis H test: *p<0.05; **p<0.01; ***p<0.001.

For total phenolic content (TPC) (**Figure 7**), microbial inoculation generally decreased TPC, except for *P.p.*, in the 13-99-1K under FLUO, which increased it by 65%. *P.p.* decreased TPC in other experimental groups for this family (64% in PHE and 62% in NAPH). *S.y.* reduced TPC by 59% under PHE in the 13-99-1K seedlings. *R.s.* also decreased TPC in the 13-99-1K family, with reductions of 59% in NAPH and significant decreases in PYR and FLUO. In the 41-65-7K half-sib family, *P.p.* decreased TPC under PHE. In controls, PHE and NAPH increased TPC in the 13-99-1K seedlings compared to untreated plants.

Welch ANOVA (**Supplementary Table S7**) indicated significant Family × Pollutant × Microorganism interaction in TPC (*p* < 0.001). Pairwise Wilcoxon test (**Supplementary Table S9**) revealed family differences. *Post hoc* tests (**Supplementary Table S11**) highlighted significant differences between control and *R.s.*, control and *S.y.*, *P.p.* and *R.s.*, and *R.s.* and *S.y.* Significant variations were also found between control and PHE, and between PHE and FLUO.

**Figure 8:**
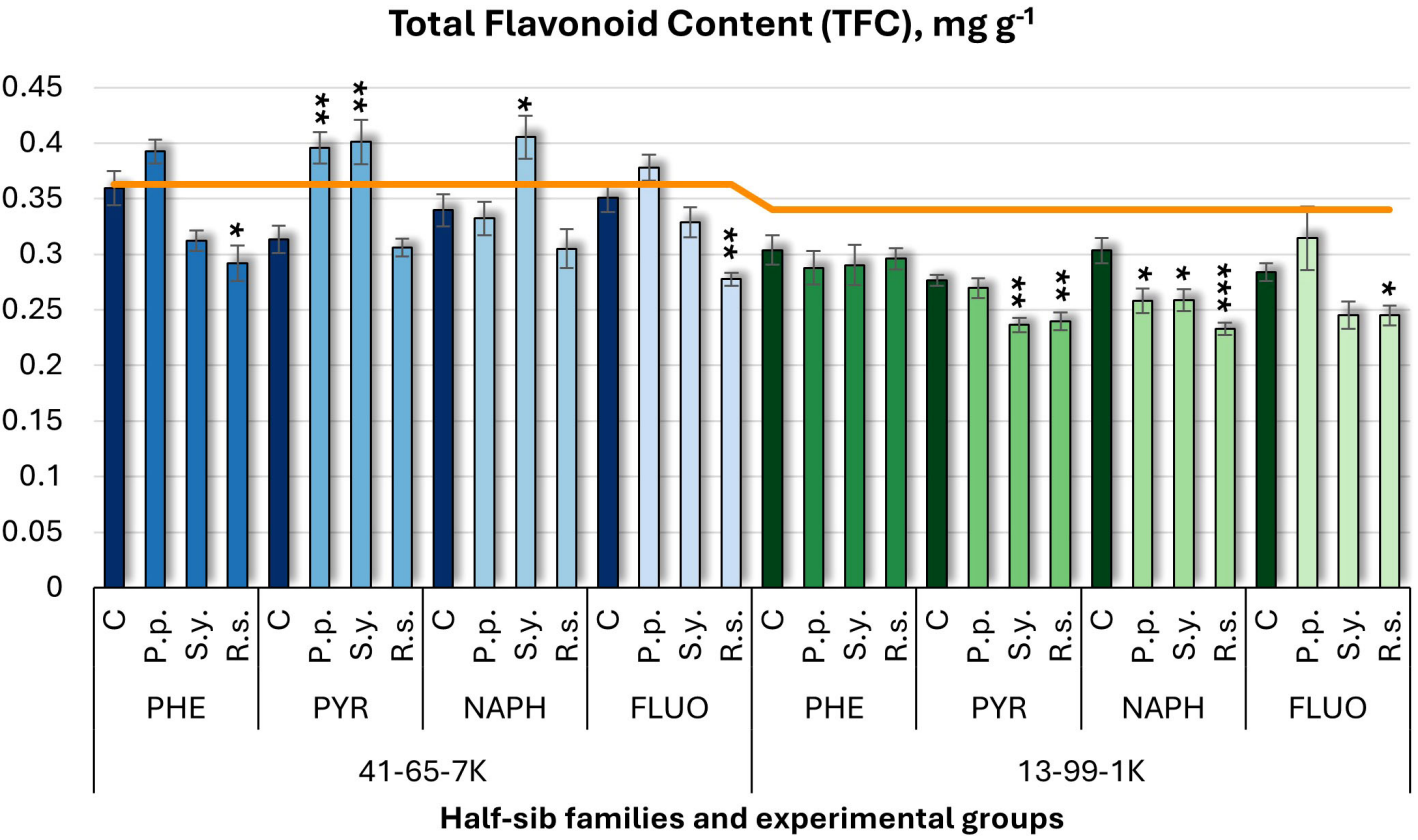
Total flavonoid content (TFC) (mg g^-1^ fresh weight) comparison in black alder half-sib families (41-65-7K and 13-99-1K) under various microbial inoculations: control (C), *Pseudomonas putida* (*P.p*.), *Sphingobium yanoikuyae* (*S.y.*), and *Rhodotorula* sp*haerocarpa* (*R.s.*), across different pollutant treatments: phenanthrene (PHE), pyrene (PYR), naphthalene (NAPH), and fluoranthene (FLUO). The orange line indicates the mean of TFC in the absence of pollutants and microbial inoculations. Each pollutant treatment includes a specific control (C), representing plants treated with that specific pollutant but without microbial inoculation by which statistical significance was assessed. Biochemical analyses were conducted on three biological replicates per treatment, with each replicate consisting of leaves pooled from at least five different plants. Data are presented as mean ± standard error (SE). Statistically significant differences from the control group were assessed using the Kruskal-Wallis H test: *p<0.05; **p<0.01; ***p<0.001.

For total flavonoid content (TFC) (**Figure 8**), *P.p.* inoculation increased TFC in the 41-65-7K half-sib family under PYR but decreased in 13-99-1K under NAPH. *S.y.* increased TFC in 41-65-7K half-sib family seedlings but decreased it in 13-99-1K under PYR and NAPH. *R.s.* consistently decreased TFC in the 41-65-7K family when grown with PHE and FLUO and in the 13-99-1K family when seedlings were treated with PYR, NAPH, and FLUO.

Welch ANOVA (**Supplementary Table S7**) revealed significant Family × Pollutant × Microorganism interaction in TFC (*p* < 0.001). Pairwise Wilcoxon test (**Supplementary Table S9**) showed family differences in TFC levels. Pairwise comparisons (**Supplementary Table S11**) highlighted significant differences between control and *R.s.*, *P.p*. and R.s., and *R.s.* and *S.y.* No significant differences for pollutant effects.

### 3.5 Antioxidant enzyme activities: catalase, superoxide dismutase, guaiacol peroxidase, ascorbate peroxidase, glutathione S-transferase, and glutathione reductase

**Figure 9:**
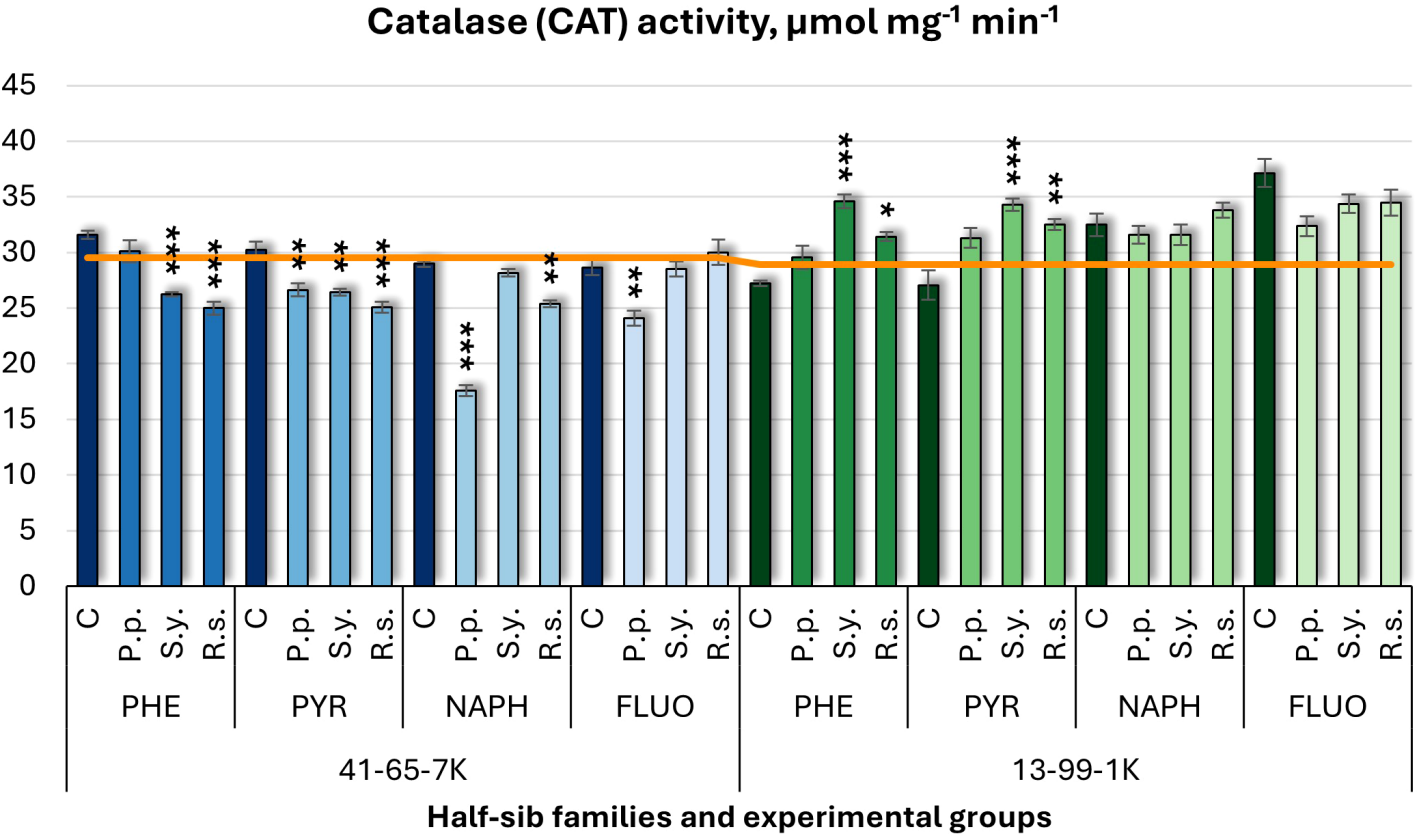
Catalase (CAT) activity (µmol mg^-1^ (protein) min^-1^) comparison in black alder half-sib families (41-65-7K and 13-99-1K) under various microbial inoculations: control (C), *Pseudomonas putida* (*P.p*.), *Sphingobium yanoikuyae* (*S.y.*), and *Rhodotorula* sp*haerocarpa* (*R.s.*), across different pollutant treatments: phenanthrene (PHE), pyrene (PYR), naphthalene (NAPH), and fluoranthene (FLUO). The orange line indicates the mean of CAT activity in the absence of pollutants and microbial inoculations. Each pollutant treatment includes a specific control (C), representing plants treated with that specific pollutant but without microbial inoculation by which statistical significance was assessed. Biochemical analyses were conducted on three biological replicates per treatment, with each replicate consisting of leaves pooled from at least five different plants. Data are presented as mean ± standard error (SE). Statistically significant differences from the control group were assessed using the Kruskal-Wallis H test: *p<0.05; **p<0.01; ***p<0.001.

For catalase (CAT) activity (**Figure 9**), microbial inoculation had differing effects on the two half-sib families. In 41-65-7K family, *P.p.* decreased CAT activity under PYR, NAPH (by 39%), and FLUO treatments. In contrast, seedlings of the 13-99-1K family generally showed increased CAT activity, with no significant changes in response to *P.p. S.y.* had opposite effects: it increased CAT in 41-65-7K half-sib family seedlings under PHE and PYR but decreased it in 13-99-1K half-sib family seedlings. *R.s.* consistently reduced CAT activity in both families under PYR, PHE, and NAPH treatments.

For CAT (**Supplementary Table S5**), significant effects were found for Family (*p* < 0.001), Pollutant (*p* = 0.017), and Microorganism (*p* = 0.002). All interaction terms, including two- and three-way ANOVA interactions, were also significant (p < 0.001). *Post hoc* analyses (**Supplementary Table S8**) revealed significant differences between families in CAT changes. Only *P.p.* significantly differed from the control group, and from both *R.s.* and *S.y.* (**Supplementary Table S10**). Tukey *post hoc* test (**Supplementary Table S13**) revealed significant differences in CAT changes between the control and FLUO, and between PYR and FLUO.

**Figure 10:**
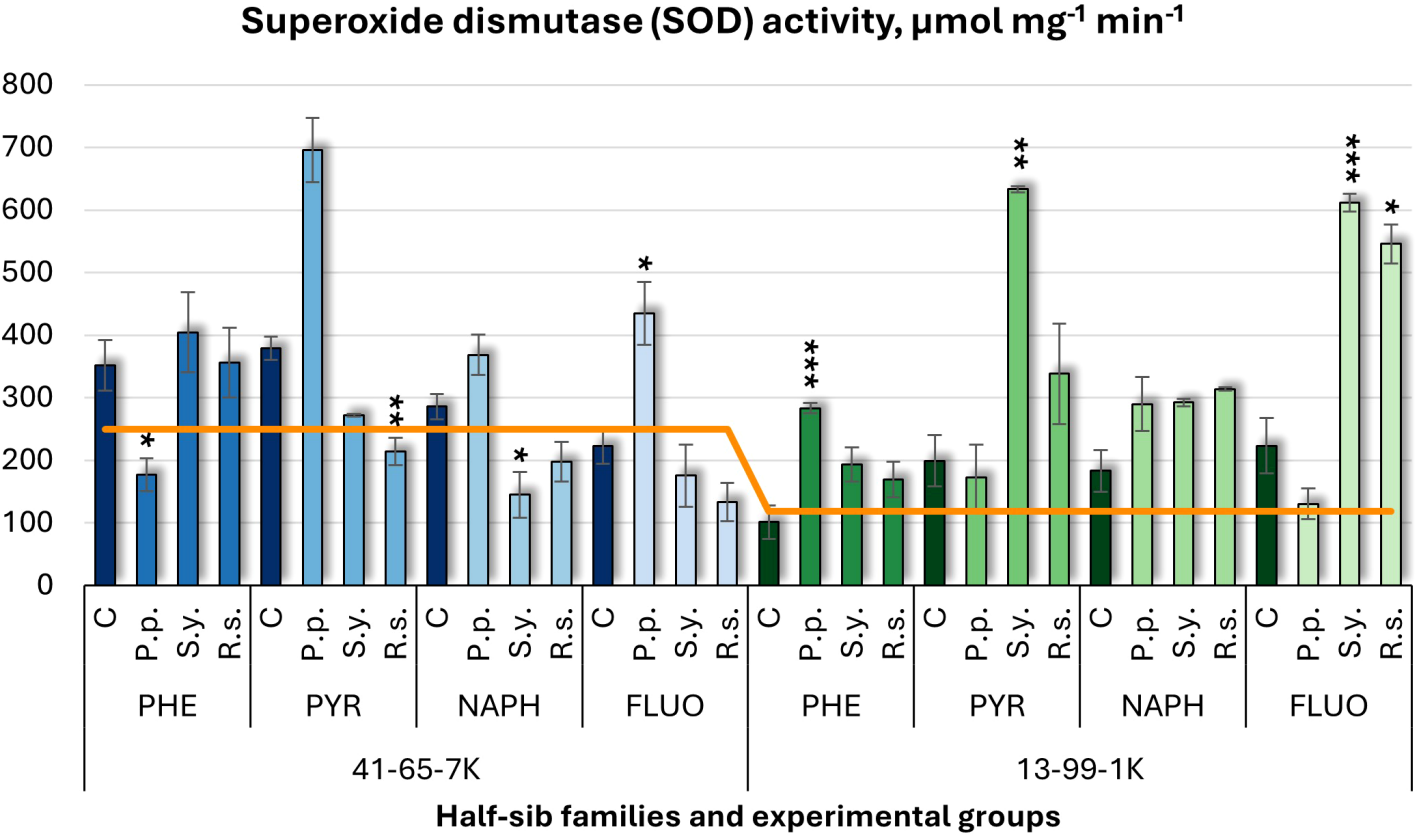
Superoxide dismutase (SOD) activity (µmol mg^-1^ (protein) min^-1^) comparison in black alder half-sib families (41-65-7K and 13-99-1K) under various microbial inoculations: control (C), *Pseudomonas putida* (*P.p*.), *Sphingobium yanoikuyae* (*S.y.*), and *Rhodotorula* sp*haerocarpa* (*R.s.*), across different pollutant treatments: phenanthrene (PHE), pyrene (PYR), naphthalene (NAPH), and fluoranthene (FLUO). The orange line indicates the mean of SOD activity in the absence of pollutants and microbial inoculations. Each pollutant treatment includes a specific control (C), representing plants treated with that specific pollutant but without microbial inoculation by which statistical significance was assessed. Biochemical analyses were conducted on three biological replicates per treatment, with each replicate consisting of leaves pooled from at least five different plants. Data are presented as mean ± standard error (SE). Statistically significant differences from the control group were assessed using the Kruskal-Wallis H test: *p<0.05; **p<0.01; ***p<0.001.

For superoxide dismutase (SOD) activity (**Figure 10**), *P.p.* increased SOD activity by 95% in the 41-65-7K half-sib family under FLUO treatment but decreased it under PHE. In the 13-99-1K family, *P.p.* increased SOD activity by 180% under PHE. *S.y*. had mixed effects: it decreased SOD activity in the 41-65-7K family under NAPH treatment but increased it by 217% in the 13-99-1K family under PYR, and by 174% under FLUO. *R.s.* reduced SOD activity in the 41-65-7K seedlings under PYR (44% decrease) but increased it by 144% in the 13-99-1K seedlings under FLUO. Untreated 13-99-1K family seedlings had lower SOD activity than untreated seedlings of the 41-65-7K family.

The Welch ANOVA (**Supplementary Table S7**) indicated significant effects of the Family × Pollutant × Microorganism interaction in SOD changes (*p* < 0.001), along with significant effects of Pollutant. The pairwise Wilcoxon test results (**Supplementary Table S14**) found significant differences between the control and PYR, and between PYR and PHE. No significant differences in SOD variation were observed between families or in the effects of microorganisms or pollutants.

**Figure 11:**
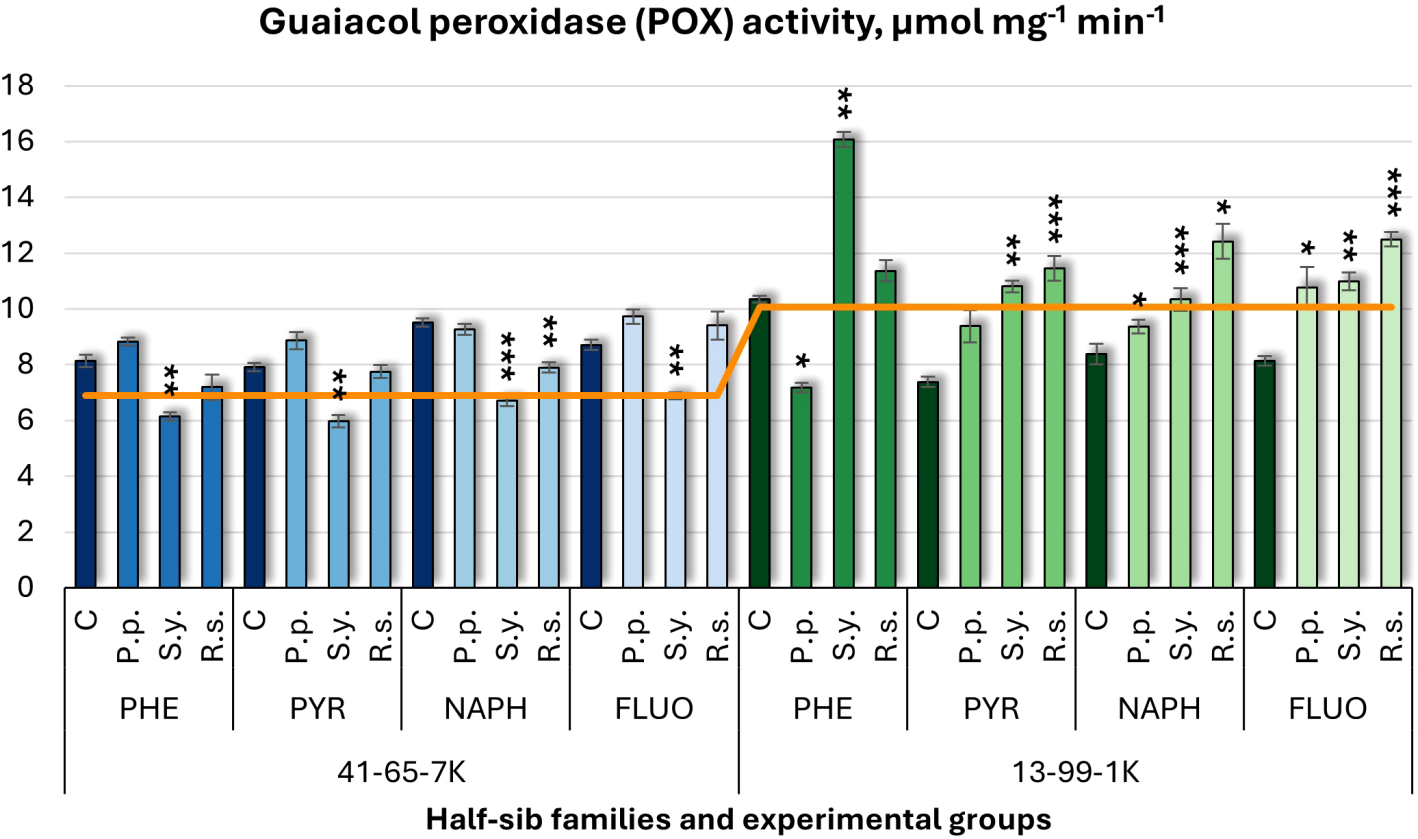
Guaiacol peroxidase (POX) activity (µmol mg^-1^ (protein) min^-1^) comparison in black alder half-sib families (41-65-7K and 13-99-1K) under various microbial inoculations: control (C), *Pseudomonas putida* (*P.p*.), *Sphingobium yanoikuyae* (*S.y.*), and *Rhodotorula* sp*haerocarpa* (*R.s.*), across different pollutant treatments: phenanthrene (PHE), pyrene (PYR), naphthalene (NAPH), and fluoranthene (FLUO). The orange line indicates the mean of POX activity in the absence of pollutants and microbial inoculations. Each pollutant treatment includes a specific control (C), representing plants treated with that specific pollutant but without microbial inoculation by which statistical significance was assessed. Biochemical analyses were conducted on three biological replicates per treatment, with each replicate consisting of leaves pooled from at least five different plants. Data are presented as mean ± standard error (SE). Statistically significant differences from the control group were assessed using the Kruskal-Wallis H test: *p<0.05; **p<0.01; ***p<0.001.

The guaiacol peroxidase (POX) activity results (**Figure 11**) followed a similar pattern to the previous enzymes. The 41-65-7K half-sib family experienced a decrease in POX activity, while the 13-99-1K half-sib family showed increased activity. *P.p.* had a statistically affected the 13-99-1K seedlings, decreasing POX activity under PHE, but increasing it in NAPH and FLUO media. *S.y.* decreased POX activity in the 41-65-7K half-sib family but increased it in the 13-99-1K family, particularly by 55% under PHE. *R.s.* decreased POX activity in the 41-65-7K seedlings in NAPH-containing medium but in the 13-99-1K seedlings under PYR, NAPH, and FLUO treatments.

Welch ANOVA (**Supplementary Table S7**) indicated significant effects of the Family × Pollutant × Microorganism interaction in POX changes (*p* < 0.001), as well as significant effects of Family. Pairwise Wilcoxon test results (**Supplementary Table S9**) revealed differences between families in POX changes. Pairwise comparisons for the Microorganism effects (**Supplementary Table S11**) highlighted significant differences between *R.s.* and *S.y*, but no significant differences were detected for POX concerning pollutant effects.

**Figure 12:**
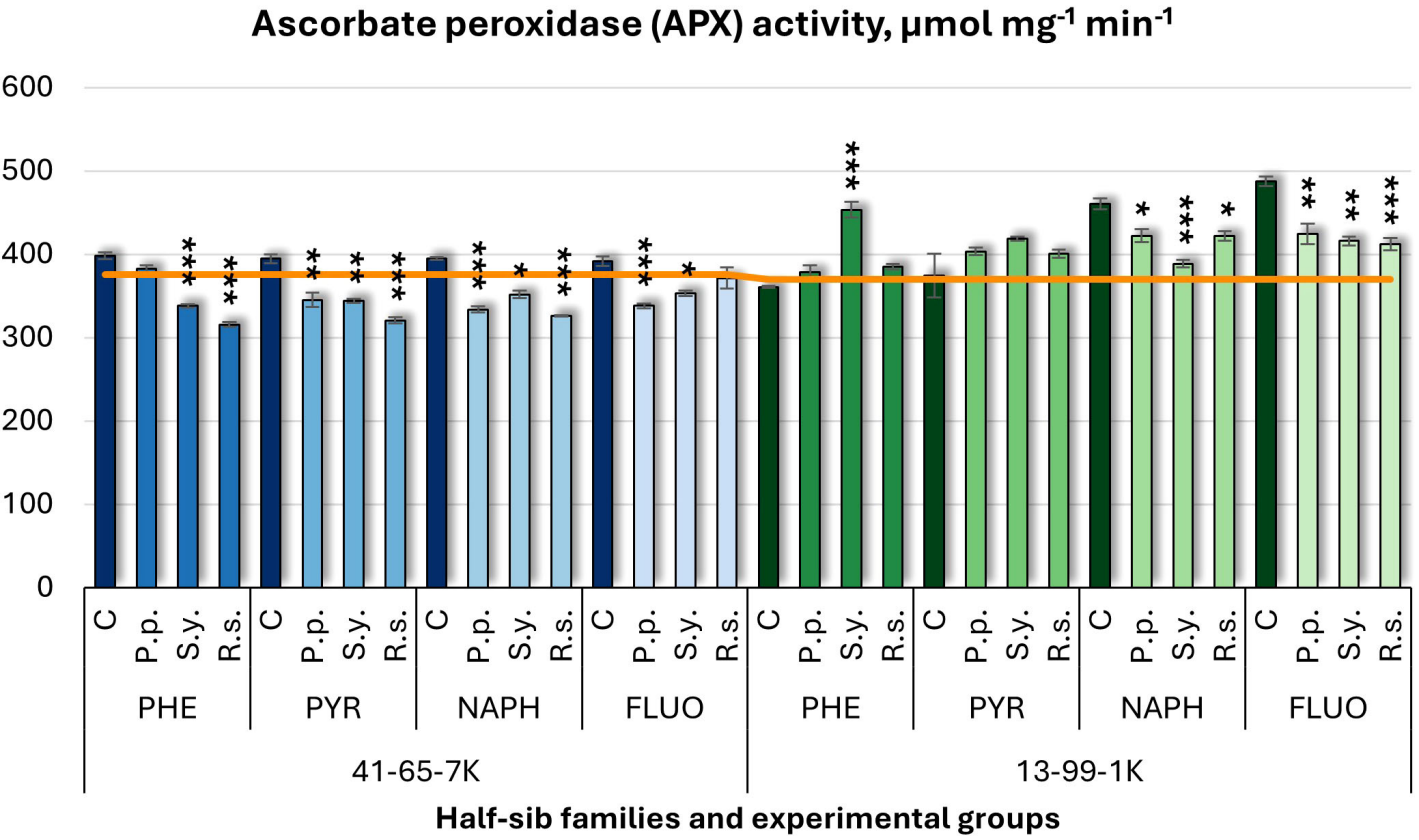
Ascorbate peroxidase (APX) activity (µmol mg^-1^ (protein) min^-1^) comparison in black alder half-sib families (41-65-7K and 13-99-1K) under various microbial inoculations: control (C), *Pseudomonas putida* (*P.p*.), *Sphingobium yanoikuyae* (*S.y.*), and *Rhodotorula* sp*haerocarpa* (*R.s.*), across different pollutant treatments: phenanthrene (PHE), pyrene (PYR), naphthalene (NAPH), and fluoranthene (FLUO). The orange line indicates the mean of APX activity in the absence of pollutants and microbial inoculations. Each pollutant treatment includes a specific control (C), representing plants treated with that specific pollutant but without microbial inoculation by which statistical significance was assessed. Biochemical analyses were conducted on three biological replicates per treatment, with each replicate consisting of leaves pooled from at least five different plants. Data are presented as mean ± standard error (SE). Statistically significant differences from the control group were assessed using the Kruskal-Wallis H test: *p<0.05; **p<0.01; ***p<0.001.

Regarding ascorbate peroxidase (APX) activity (**Figure 12**), bacterial inoculation decreased activity, with one exception. *P.p.* decreased APX activity in the 41-65-7K half-sib family when seedlings were exposed to PYR, NAPH, and FLUO and in the 13-99-1K family when seedlings grown in NAPH and FLUO-containing media. *S.y.* consistently decreased APX activity in the 41-65-7K family but had a dual effect in 13-99-1K half-sib family seedlings: APX activity increased when seedlings were grown in PHE-containing medium but decreased when grown in NAPH and FLUO media. *R.s.* also decreased APX in both families: in the 41-65-7K family under PHE, PYR, and NAPH treatments and in the 13-99-1K family under NAPH and FLUO treatments. Notably, NAPH and FLUO treatments led to significantly higher APX activity in the 13-99-1K control group than the untreated plants.

Welch ANOVA (**Supplementary Table S7**) indicated significant effects of the Family × Pollutant × Microorganism interaction in APX levels (*p* < 0.001) and Family effects, as shown by the pairwise Wilcoxon test (**Supplementary Table S9**). Pairwise comparisons (**Supplementary Table S11**) highlighted significant differences in APX between control and *S.y.*, and control and *R.s.* No significant differences were detected for APX concerning pollutant effects.

**Figure 13:**
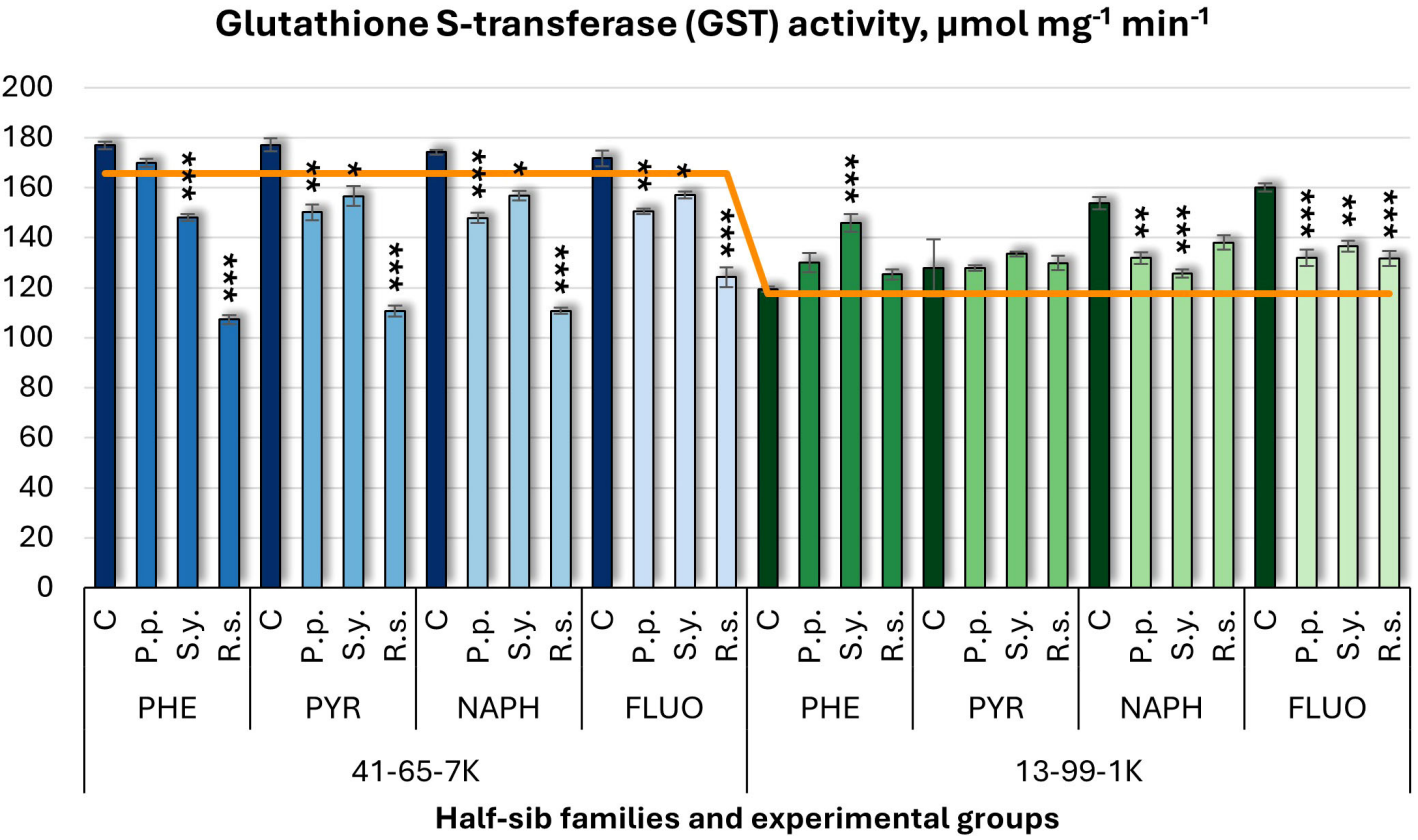
Glutathione S-transferase (GST) activity (µmol mg^-1^ (protein) min^-1^) comparison in black alder half-sib families (41-65-7K and 13-99-1K) under various microbial inoculations: control (C), *Pseudomonas putida* (*P.p*.), *Sphingobium yanoikuyae* (*S.y.*), and *Rhodotorula* sp*haerocarpa* (*R.s.*), across different pollutant treatments: phenanthrene (PHE), pyrene (PYR), naphthalene (NAPH), and fluoranthene (FLUO). The orange line indicates the mean of GST activity in the absence of pollutants and microbial inoculations. Each pollutant treatment includes a specific control (C), representing plants treated with that specific pollutant but without microbial inoculation by which statistical significance was assessed. Biochemical analyses were conducted on three biological replicates per treatment, with each replicate consisting of leaves pooled from at least five different plants. Data are presented as mean ± standard error (SE). Statistically significant differences from the control group were assessed using the Kruskal-Wallis H test: *p<0.05; **p<0.01; ***p<0.001.

Similar to APX, glutathione S-transferase (GST) activity (**Figure 13**) was influenced by microbial inoculation. *P.p.* decreased GST activity in the 41-65-7K half-sib family under PYR, NAPH, and FLUO treatments, and in the 13-99-1K family under NAPH and FLUO. *S.y.* showed a dual effect: it decreased GST activity in the seedlings of half-sib family 41-65-7K across all pollutants, while it increased GST in the 13-99-1K family under PHE but decreased it under NAPH and FLUO. *R.s.* decreased GST activity in the 41-65-7K family under all pollutants, with reductions ranging from 28% to 39%, while in the 13-99-1K family, *R.s.* only decreased GST activity under FLUO. Untreated plants of the 13-99-1K family had higher GST activity than the control groups under NAPH and FLUO.

Welch ANOVA (**Supplementary Table S7**) revealed a significant Family × Pollutant × Microorganism interaction effect on GST levels (p < 0.001). *Post hoc* analysis (**Supplementary Table S8**) showed significant differences between families in GST changes. For Microorganism effects (**Supplementary Table S10**), all inoculated groups differed significantly from the control, with *R.s.* also differing from both *P.p.* and *S.y.* Significant differences were observed in all comparisons with the control, indicating a consistent response to pollutant exposure, using the pairwise Wilcoxon test (**Supplementary Table S14**).

**Figure 14:**
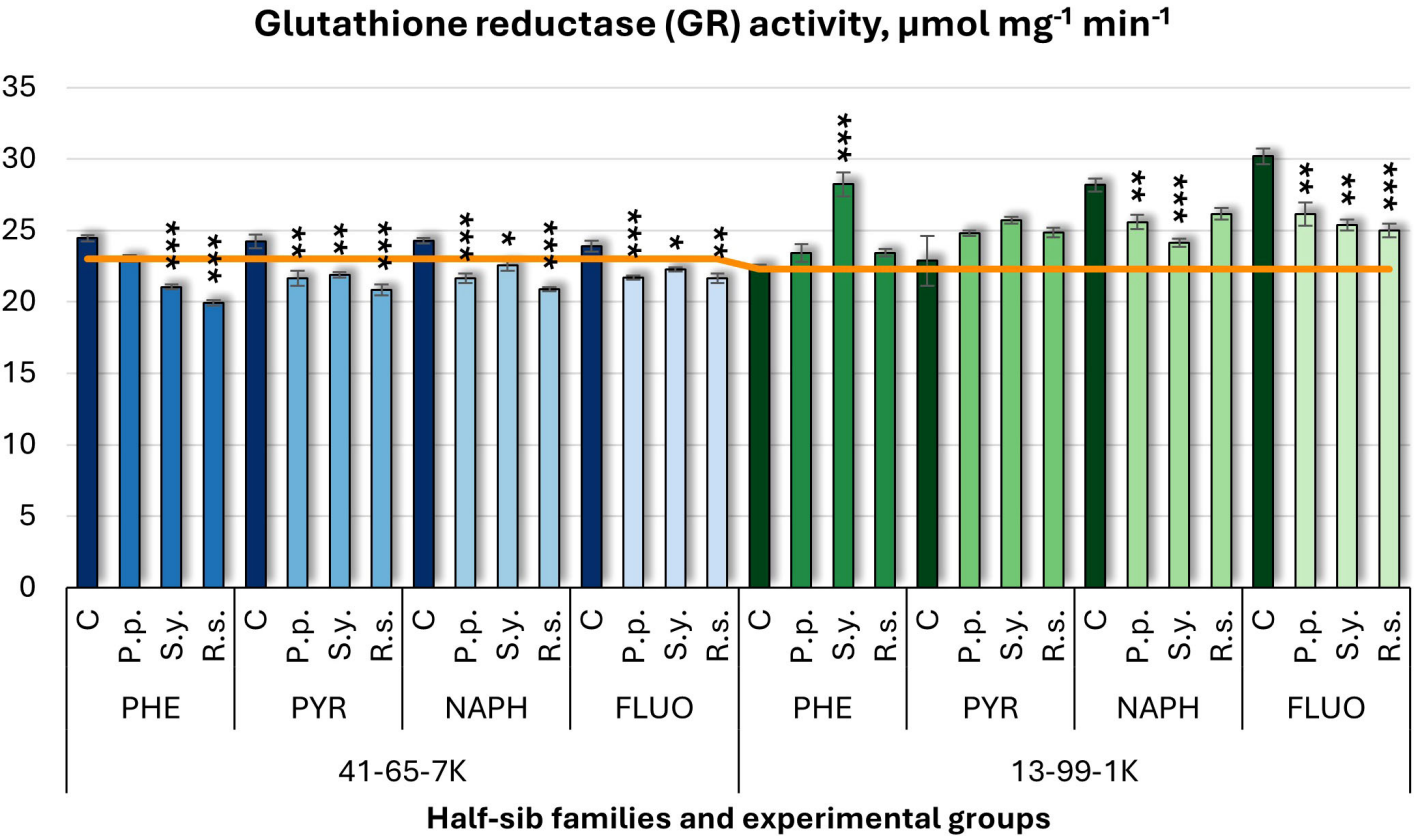
Glutathione reductase (GR) activity (µmol mg^-1^ (protein) min^-1^) comparison in black alder half-sib families (41-65-7K and 13-99-1K) under various microbial inoculations: control (C), *Pseudomonas putida* (*P.p*.), *Sphingobium yanoikuyae* (*S.y.*), and *Rhodotorula* sp*haerocarpa* (*R.s.*), across different pollutant treatments: phenanthrene (PHE), pyrene (PYR), naphthalene (NAPH), and fluoranthene (FLUO). The orange line indicates the mean of GR activity in the absence of pollutants and microbial inoculations. Each pollutant treatment includes a specific control (C), representing plants treated with that specific pollutant but without microbial inoculation by which statistical significance was assessed. Biochemical analyses were conducted on three biological replicates per treatment, with each replicate consisting of leaves pooled from at least five different plants. Data are presented as mean ± standard error (SE). Statistically significant differences from the control group were assessed using the Kruskal-Wallis H test: *p<0.05; **p<0.01; ***p<0.001.

Glutathione reductase (GR) activity results (**Figure 14**) displayed similar decreasing patterns to APX and GST. *P.p.* decreased GR activity in the 41-65-7K half-sib family under PYR, NAPH, and FLUO treatments and in the 13-99-1K half-sib family under NAPH and FLUO. *S.y.* decreased GR activity in all pollutant groups in the 41-65-7K family but increased it in the 13-99-1K family under PHE, with a decrease under NAPH and FLUO. *R.s.* consistently decreased GR activity in the 41-65-7K seedlings across all pollutants, and only under FLUO in the 13-99-1K family seedlings. Control groups in the 13-99-1K family exhibited higher GR activity under NAPH and FLUO compared to untreated plants.

Welch ANOVA (**Supplementary Table S7**) indicated significant effects of the Family × Pollutant × Microorganism interaction on all tested parameters (CHL, TPC, TFC, APX, POX, SOD, CAR, and SS), including GR (*p* < 0.001). Pairwise Wilcoxon test (**Supplementary Table S9**) revealed differences between families n GR changes. *Post hoc* analysis for Microorganism effects (**Supplementary Table S10**) revealed significant differences between control and *R.s.*, and control and *S.y.* No significant differences were detected for GR concerning pollutant effects.

### 3.6 Principal component analysis of microorganism, family, and pollutant impact on all indicators

**Figure 15:**
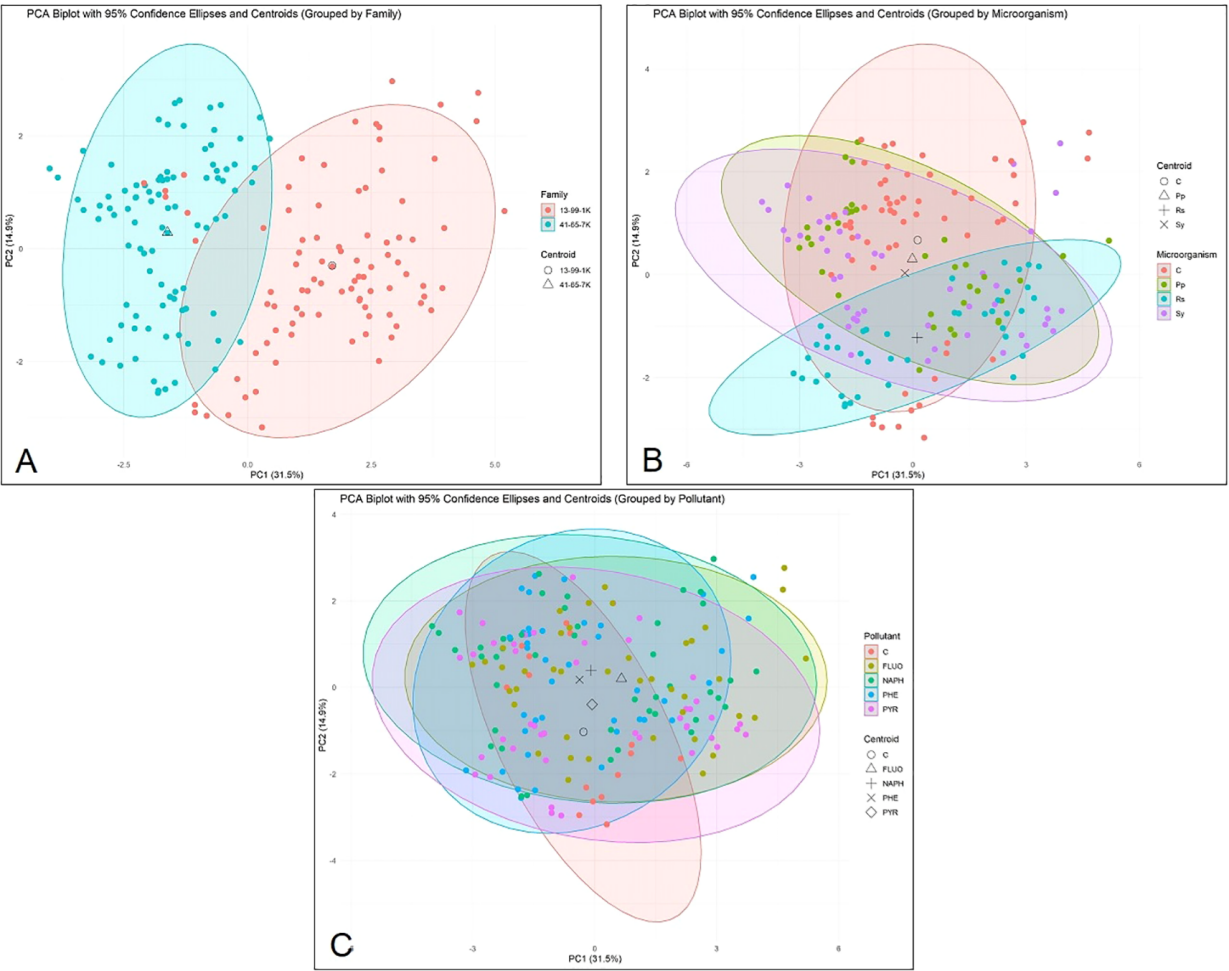
Principal component analysis (PCA) biplot of black alder seedling data. The ellipses in each plot represent 95% confidence intervals for the respective groups, with centroids indicated by distinct shapes, as shown in the legend. **(A)** PCA biplot for Family, comparing two families: 13-99-1K and 41-65-7K. **(B)** PCA biplot for Microorganisms, showing the response of black alder seedlings to different microbial treatments: C (control, not inoculated), Pp (*Pseudomonas putida*), Sy (*Sphingobium yanoikuayae*), and Rs (*Rhodotorula* sp*haerocarpa*). **(C)** PCA biplot for Pollutants, illustrating the effects of various pollutants on black alder seedlings: C (control, no pollutant), PHE (phenanthrene), PYR (pyrene), NAPH (naphthalene), and FLUO (fluoranthene). The x and y axes represent the first two principal components (PC1 and PC2), with the percentage of variance explained by each principal component indicated in the axis labels.

In **Figure 15A** PC1 (principal component) (31.5%) explains the most variance, meaning it captures the strongest differentiation between the two genetic families, while PC2 (14.9%) explains less variance but still provides meaningful separation. Despite overlapping individuals, the centroids suggest a significant genetic distinction between families, based on the indicators tested. The spread and overlap might indicate that some biochemical and growth markers are shared between families, while others drive differentiation.

In **Figure 15B** PC1 and PC2 represent 46.4% of the total variance (the same as in other two graphs, as the same data set was used). The clusters, representing different microorganism treatment groups partially overlap, meaning the biochemical and growth markers do not completely separate treatments, but some differentiation exists. The untreated (Control) group has a broad spread, suggesting high variability in untreated seedlings. *R.s*. is somewhat concentrated but overlaps significantly with other treatments. *P.p.* and *S.y.* overlap but extend uniquely, meaning they might induce different but not entirely exclusive changes. Each treatment has a distinct centroid, confirming that their average biochemical and growth profiles are different. The distinct clustering and centroid position of *R.s.* might indicate a stronger effect on specific biochemical or growth traits compared to other treatments.

**Figure 15C** represents the same data set differentiated based on the tested PAHs. The clusters overlap significantly, suggesting the biochemical and growth markers do not completely separate treatments. The untreated (Control) group has the most distinct spread, suggesting differences in indicator variability in untreated seedlings. However, while the clusters of the pollutants overlap, the average individual, as represented by centroids for each cluster, are separate, indicating that each tested PAH induces distinct average biochemical and growth changes.

Overall, the PCA data suggests that, based on the indicators tested, all three factors (microorganism strain, pollutant type, and half-sib (genetic) family) impact the distribution of the results and are therefore important determinants of the final biochemical and growth responses of black alder seedlings.

## 4 Discussion

This research aimed to elucidate the impact of selected microorganisms on mitigating the phytotoxic effects of various PAHs in black alder (*Alnus glutinosa*). Our hypothesis posited that these microbial strains - *Pseudomonas putida* (*P.p.*), *Sphingobium yanoikuyae* (*S.y.*), and *Rhodotorula* sp*haerocarpa* (*R.s.*) *-* could alleviate damage by reducing oxidative stress and improving plant growth under PAH exposure. The data on the impact of the selected microbes on alder was previously reported, showcasing that inoculation could lead to increased growth and reduced lipid peroxidation (Striganavičiūtė et al., 2024b). The data presented here indicate a complex, context-dependent pattern of plant-microbe interactions in regard to the 4 tested PAHs. The hypothesis was supported in some cases but challenged in others, depending on a triad of factors: alder genotype, microbial strain, and PAH type (naphthalene, pyrene, phenanthrene or fluorene). These findings are significant for understanding the dynamics of microbe-assisted phytoremediation and their practical implications.

Growth and biochemical responses were observed to be highly variable, reflecting a multifactorial interplay. However, several key types of impact could also be discerned. When evaluating the conglomerate of biochemical markers, it’s also important to look at plant growth patterns. As such, groups exhibiting reduced growth and elevated MDA levels, expose a level of stress the plant cannot deal with, indicating a highly toxic environment, which has been noted for PAHs and other plants (Alkio et al., 2005; Molina and Segura, 2021). Opposingly, some groups exhibited either elevated (trending, but non-significantly) or unchanging growth parameters, with lower MDA levels and reduced antioxidative enzyme activity. This indicates that the treatment is successfully enhancing cellular health and reducing oxidative stress without compromising the plant’s growth potential. This could be due to several mechanisms like a shift toward a low-stress state. The treatment might be effectively reducing oxidative stress and damage, allowing the plant cells to allocate more energy and resources toward growth processes rather than repair (Grover et al., 2011; Tiwari et al., 2016; Ikram et al., 2018). It could also indicate a treatment’s dual role in stress mitigation and growth promotion: the inoculation may be providing benefits beyond stress reduction, potentially by enhancing nutrient uptake, water use efficiency, or hormone regulation, all of which could directly or indirectly support growth. This was previously achieved for both *P. putida* and *S. yanoikuyae* in chickpeas and willows respectively (Tiwari et al., 2016; Zeng et al., 2020). Overall, this pattern suggests the alder might be adjusting its physiological baseline to thrive in a stressful environment, potentially due to the treatment’s effects (induced resistance). Such an adjustment could make the plant more resilient over time, with minimal need for stress-related adaptations (Choudhary et al., 2007; Eyles et al., 2010; Fontana et al., 2021).

Results showed that in some cases, like family 13-99-1K treated with PHE and *S.y.*, growth remained steady while MDA levels dropped, but antioxidant enzyme levels went up. This could indicate that the treatment is actively enhancing the plant’s defense mechanisms against oxidative stress, as was noted by Ikram et al. (2018) in their research on the *Penicillium roqueforti* inoculated wheat grown on heavy metal contaminated soil. This scenario might imply an enhanced antioxidant defense system. This enhancement could preemptively counter oxidative stress, therefore the plant might be in a “primed” state, ready to handle potential stress more effectively (Conrath et al., 2001). Similar results were reported in another study with wheat and also Norway spruce (Lastochkina et al., 2020; Mageroy et al., 2020). Another observed interplay was in family 41-65-7K treated with PHE and *P.p.* bacterium. MDA levels went up, as did chlorophyll *a/b* ratio, while SS and TPC levels dropped and at the same time shoot height trended upwards with a negligible enzymatic response. It suggests that, although oxidative stress is present, the plant is adapting positively to the stress. The increased chlorophyll *a/b* ratio indicates improved photosynthetic efficiency under stress, contributing to growth as was noted in studies done with soybean and sunflower inoculated with *Aspergillus japonicus* (Ismail et al., 2018). However, the concurrent drop in soluble sugars and phenols, alongside stable antioxidant enzymes (except for reduced SOD), implies that the plant is diverting resources toward growth rather than sustaining its usual metabolic defenses (Ismail et al., 2018), possibly because the microorganisms are assisting in PAH detoxification based on the findings of Mitra et al. (2020) and Cunliffe and Kertesz (2006) on the *S.y.* strain and Weyens et al. (2010) on *P.p.* species/strain. This could be due to the need to prioritize recovery and adaptation to the PAH stress, aided by microorganisms. Alternatively, the increase in MDA might not directly correlate with overall damage but could reflect an adaptive signaling response, where some oxidative stress is used as a signal to activate growth pathways rather than merely a marker of injury. This has been observed in model species *Arabidopsis thaliana* and mentioned in multiple reviews (Liu et al., 2009; Gill and Tuteja, 2010; Sharma et al., 2012; Isah, 2019).

Further on, observing the data collectively, a trend towards chlorophyll *a/b* ratio and carotenoid content going down can be noted. This was mentioned previously by Pliūra et al. (2019) among others, where it was noted that a reduction in the plant’s photosynthetic efficiency, often implies plant stress or cellular damage (Sarker and Oba, 2018; Iqbal et al., 2019). Similarly, TPC and TFC levels usually dropped concurrently as well. Phenolics usually relate to a stress response. In our study, usually a reduced stress at that, as growth remained unchanged (Selmar and Kleinwächter, 2013; Zaynab et al., 2018; Wallis and Galarneau, 2020). Additionally, soluble sugar content was also affected negatively in most cases and in few instances where it increased, growth usually either statistically significantly decreased or trended towards reduction. As these sugars play key roles in energy storage, osmoregulation, and stress responses (Rosa et al., 2009; Gangola and Ramadoss, 2018), the interpretation of these changes alone is difficult. However, reduced sugar content can suggest a significant diversion of carbon resources to stress response pathways (Rosa et al., 2009).

Overall, the differential impact on black alder genotypes further complicates the interpretation. Genetic variability in antioxidant capacity, phenolic production, and stress signaling pathways likely underlies the observed genotype-specific responses, as presented by Tervahauta et al. (2009) and Iqbal et al. (2019) examining the response of different birch and soybeans genotypes on abiotic stresses respectively. One genotype may exhibit a more robust inherent ability to synthesize protective phenolics and activate antioxidative enzymes, thereby mitigating PAH stress more effectively. This genotype dependency is aligned with research demonstrating the role of plant genetic background in shaping responses to abiotic stress, including heavy metals and organic pollutants (Isah, 2019).

These findings have considerable implications for designing microbe-assisted phytoremediation systems. The effectiveness of specific microorganisms in helping with the phytotoxicity of specific PAHs highlights the potential for employing microorganisms based on site contamination type (Sakshi and Haritash, 2020; Shi et al., 2022; Davletgildeeva and Kuznetsov, 2024). Furthermore, according to Ghosal et al. (2016) and Ahmad (2021) this also implies that for sites contaminated with multiple PAHs bioremediation strategies should consider microbial consortia. Moreover, the variable responses between alder genotypes suggest that selecting appropriate plant genotypes, along with tailored microbial inoculants, could optimize phytoremediation outcomes. The ability to strategically match microbial species with compatible plant genotypes for specific contaminant profiles represents a promising direction for ecological restoration and contamination management.

However, while the results are promising, the study’s limitations should be noted. The hydroponic system does not fully capture the complexity of soil environments, nor do the controlled conditions of the growth chamber represent the environment of the open ecosystem (Migeon et al., 2012; Cao et al., 2018). Additionally, the short experimental duration may not reflect the long-term dynamics of plant-microbe-PAH interactions (Wieser et al., 2003; Rosales-Castro et al., 2015; Turfan et al., 2018; Lachowicz et al., 2019). Future studies should focus on soil-based experiments, explore longer-term plant responses, and investigate the use of microbial consortia. Research should also prioritize understanding the genetic mechanisms underlying genotype-specific responses to better inform phytoremediation strategies. Despite these limitations, our key findings underscore the potential of harnessing microbial-plant partnerships for sustainable remediation of PAH-contaminated sites.

In conclusion, our study demonstrates that specific microorganisms can influence the growth and biochemical responses of *Alnus glutinosa* seedlings exposed to PAHs, but the effects vary significantly depending on the genotype of the plant, microbial strain, and the type of PAH contaminant. For example, treatment with *Sphingobium yanoikuyae* strain in family 13-99-1K exposed to phenanthrene reduced MDA levels, and increased antioxidant enzyme activity, indicating enhanced stress resilience. Conversely, in family 41-65-7K treated with *Pseudomonas putida* strain and phenanthrene, oxidative stress markers (MDA) increased, while growth improved, suggesting that the plant may have diverted resources towards growth despite the presence of stress. These results support the potential of utilizing targeted microbial inoculants in phytoremediation, while emphasizing the importance of selecting appropriate plant genotypes and microbial strains based on the specific environmental conditions and contaminant types.

## Data Availability

The original contributions presented in the study are included in the article/**Supplementary Material**. Further inquiries can be directed to the corresponding author.
