# Supplementary material for: Harnessing microbial allies: enhancing black alder resilience to PAH stress through microbial symbiosis
Source: Front Plant Sci. 2025 May 8;16:1552258. doi: 10.3389/fpls.2025.1552258 (PMC12096139; doi:10.3389/fpls.2025.1552258)
Supplement: Supplementary file 1 [file DataSheet1.docx]

Supplementary Material

**
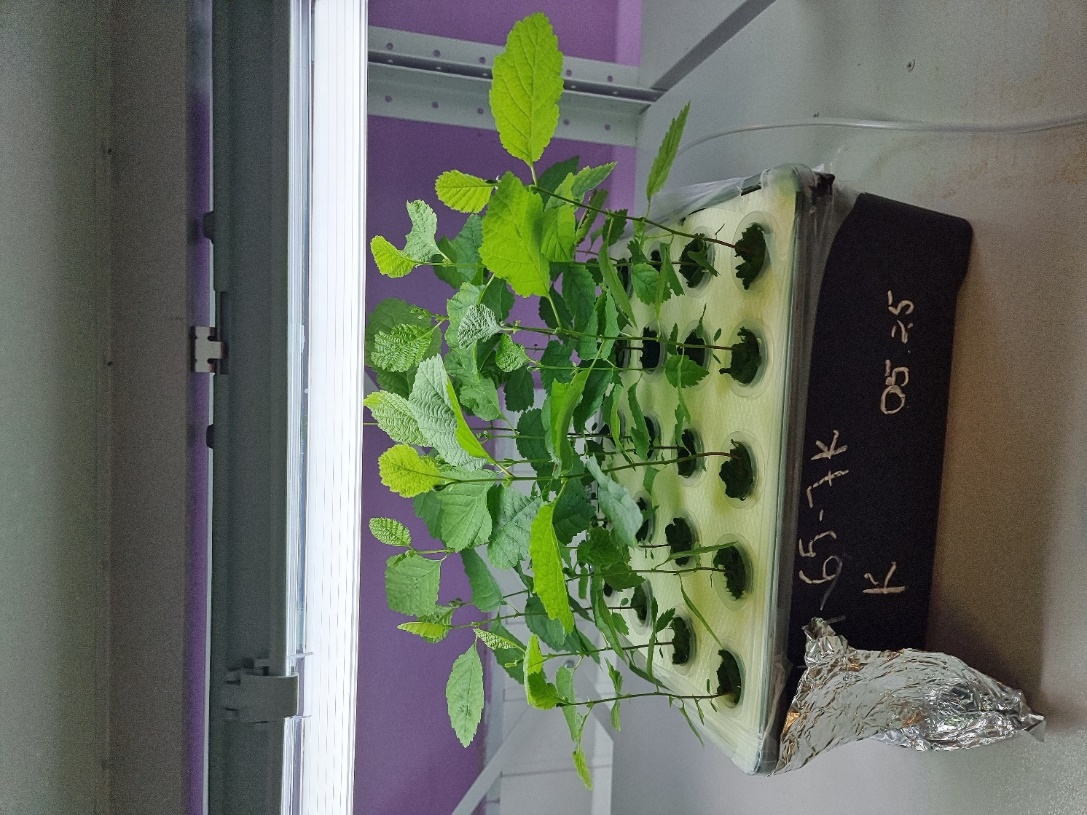
**

**Figure S1.** Black alder half-sib family 42-65-7K seedlings growing in a plastic box within the growth chamber. The box contained Hoagland nutrient solution supplemented with specific polycyclic aromatic hydrocarbons (PAHs). An air pump supplied aeration through a connected hose to ensure proper oxygenation of the solution.

**Table S1.** Levene’s Test for homogeneity of variances for various parameters, with group source and value of Df – 31.

| Parameter | F value | Pr(>F) |
| --- | --- | --- |
| Shoot | 0.8914 | 0.6339 |
| Root | 0.8442 | 0.7014 |
| MDA | 1.4458 | 0.07973 |
| CHL | 3.481 | **3.433e-07 ***** |
| TPC | 2.0604 | **0.002639** |
| TFC | 1.7156 | **0.01947** |
| APX | 1.6961 | **0.02169** |
| CAT | 0.9918 | 0.4884 |
| POX | 2.3068 | **0.000584** |
| GR | 1.7664 | **0.01467** |
| GST | 1.2655 | 0.1818 |
| SOD | 1.9147 | **0.006265** |
| CAR | 2.8816 | **1.548e-05** |
| SS | 2.5672 | **0.0001141** |

**Table S2.** Detailed Three-Way ANOVA Results for Shoot Growth. Significance levels are indicated as ******* for 𝑝<0.001.

| **Source** | **Sum Sq** | **Df** | **F-value** | **p-value** |
| --- | --- | --- | --- | --- |
| Family | 731 | 1 | 3.298 | 0.0717 |
| Pollutant | 5313 | 3 | 7.984 | **6.28e-05 ***** |
| Microorganism | 1488 | 3 | 2.235 | 0.0871 |
| Family × Pollutant | 780 | 3 | 1.172 | 0.3230 |
| Family × Microorganism | 946 | 3 | 1.421 | 0.2395 |
| Pollutant × Microorganism | 1648 | 9 | 0.825 | 0.5940 |
| Family × Pollutant × Microorganism | 3043 | 9 | 1.524 | 0.1458 |
| Residuals | 29059 | 131 |  |  |

**Table S3.** Detailed Three-Way ANOVA Results for Root length. Significance levels are indicated as ******* for 𝑝<0.001.

| **Source** | **Sum Sq** | **Df** | **F-value** | **p-value** |
| --- | --- | --- | --- | --- |
| Family | 15907 | 1 | 18.707 | **3.00e-05 ***** |
| Pollutant | 23073 | 3 | 9.045 | **1.74e-05 ***** |
| Microorganism | 937 | 3 | 0.367 | 0.7768 |
| Family × Pollutant | 6085 | 3 | 2.385 | 0.0721 |
| Family × Microorganism | 1442 | 3 | 0.565 | 0.6388 |
| Pollutant × Microorganism | 3858 | 9 | 0.504 | 0.8694 |
| Family × Pollutant × Microorganism | 10321 | 9 | 1.349 | 0.2182 |
| Residuals | 111392 | 131 |  |  |

**Table S4.** Detailed Three-Way ANOVA Results for MDA. Significance levels are indicated as ******* for 𝑝<0.001.

| **Source** | **Sum Sq** | **Df** | **F-value** | **p-value** |
| --- | --- | --- | --- | --- |
| Family | 9121 | 1 | 75.398 | **1.33e-14***** |
| Pollutant | 7112 | 3 | 19.596 | **1.48e-10 ***** |
| Microorganism | 32665 | 3 | 90.006 | **< 2e-16 ***** |
| Family × Pollutant | 14949 | 3 | 41.190 | **< 2e-16 ***** |
| Family × Microorganism | 9321 | 3 | 25.684 | **3.93e-13 ***** |
| Pollutant × Microorganism | 10437 | 9 | 9.586 | **3.81e-11 ***** |
| Family × Pollutant × Microorganism | 21416 | 9 | 19.670 | **< 2e-16 ***** |
| Residuals | 15848 | 131 |  |  |

**Table S5.** Detailed Three-Way ANOVA Results for CAT. Significance levels are indicated as ******* for 𝑝<0.001.

| **Source** | **Sum Sq** | **Df** | **F-value** | **p-value** |
| --- | --- | --- | --- | --- |
| Family | 972.9 | 1 | 207.460 | **< 2e-16 ***** |
| Pollutant | 49.4 | 3 | 3.511 | **0.01719 *** |
| Microorganism | 75.5 | 3 | 5.367 | **0.00162 **** |
| Family × Pollutant | 136.3 | 3 | 9.691 | **8.03e-06 ***** |
| Family × Microorganism | 256.3 | 3 | 18.219 | **6.11e-10 ***** |
| Pollutant × Microorganism | 199.6 | 9 | 4.729 | **1.89e-05 ***** |
| Family × Pollutant × Microorganism | 492.9 | 9 | 11.677 | **2.43e-13 ***** |
| Residuals | 614.3 | 131 |  |  |

**Table S6.** Detailed Three-Way ANOVA Results for GST. Significance levels are indicated as ******* for 𝑝<0.001.

| **Source** | **Sum Sq** | **Df** | **F-value** | **p-value** |
| --- | --- | --- | --- | --- |
| Family | 9962 | 1 | 169.928 | **< 2e-16 ***** |
| Pollutant | 2103 | 3 | 11.960 | **5.68e-07 ***** |
| Microorganism | 25882 | 3 | 147.163 | **< 2e-16 ***** |
| Family × Pollutant | 853 | 3 | 4.853 | **0.00311 **** |
| Family × Microorganism | 19579 | 3 | 111.321 | **< 2e-16 ***** |
| Pollutant × Microorganism | 3697 | 9 | 7.007 | **3.19e-08 ***** |
| Family × Pollutant × Microorganism | 5849 | 9 | 11.086 | **9.79e-13 ***** |
| Residuals | 7680 | 131 |  |  |

**Table S7.** Welch ANOVA Results for different parameters, where factor is Family × Pollutant × Microorganism; and num df is 31.

| Parameter | F value | num df | Denom df | p-value |
| --- | --- | --- | --- | --- |
| CHL | 55.938 | 31.000 | 41.039 | **< 2.2e-16** |
| TPC | 88.579 | 31.000 | 39.595 | **< 2.2e-16** |
| TFC | 17.285 | 31.000 | 40.204 | **4.778e-15** |
| APX | 85.42 | 31.000 | 39.773 | **2.2e-16** |
| POX | 61.226 | 31.000 | 40.392 | **2.2e-16** |
| GR | 62.778 | 31.000 | 40.697 | **<2.2e-16** |
| SOD | 142.34 | 31.000 | 39.587 | **< 2.2e-16** |
| CAR | 338.18 | 31.000 | 40.081 | **< 2.2e-16** |
| SS | 78.174 | 31.000 | 40.225 | **< 2.2e-16** |

**Table S8**. Detailed Post Hoc Analysis for Family effects (13-99-1K, and 41-65-7K) on different parameters, with Df value 131.

| Parameter | Estimate | SE | Df | t.ratio | p.value |
| --- | --- | --- | --- | --- | --- |
| Shoot | -4.01 | 2.44 | 131 | -1.645 | 0.1023 |
| Root | -20.2 | 4.77 | 131 | -4.227 | **<0.0001** |
| MDA | 12 | 1.8 | 131 | 6.664 | **<0.0001** |
| CAT | 5.2 | 0.354 | 131 | 14.695 | **<.0001** |
| GST | -15.6 | 1.25 | 131 | -12.453 | **<.0001** |

Table S9. Pairwise Wilcoxon Test Results for (Family).

| Parameter | p-value |
| --- | --- |
| CHL | **4.6e-07** |
| TPC | **2.3e-07** |
| TFC | **2.5e-14** |
| APX | **2e-16** |
| POX | **9.7e-13** |
| GR | **2.1e-15** |
| SOD | 0.81 |
| CAR | **<2e-16** |
| SS | **<2e-16** |

**Table S10**. Post Hoc Analysis for Microorganism Effects on different parameters, with Df value of 131.

| Parameter | Contrast | Estimate | SE | t.ratio | p.value |
| --- | --- | --- | --- | --- | --- |
| Shoot growth | C – Pp | 2.079 | 3.57 | 0.582 | 0.9374 |
|  | C – Rs | 0.439 | 3.41 | 0.715 | 0.8911 |
|  | C – Sy | -5.848 | 3.23 | -.1813 | 0.2720 |
|  | Pp – Rs | 0.359 | 3.65 | 0.098 | 0.9997 |
|  | Pp – Sy | -7.927 | 3.48 | -2.280 | 0.1079 |
|  | Rs - Sy | -8.286 | 3.31 | -2.503 | 0.0640 |
| Root length | C – Pp | 4.890 | 7.0 | 0.699 | 0.8973 |
|  | C – Rs | 4.993 | 6.68 | 0.747 | 0.8776 |
|  | C – Sy | 3.430 | 6.32 | 0.543 | 0.9483 |
|  | Pp – Rs | 0.103 | 7.14 | 0.014 | 1.0000 |
|  | Pp – Sy | -1.460 | 6.81 | -0.215 | 0.9965 |
|  | Rs - Sy | -1.563 | 6.48 | 0.241 | 0.9950 |
| MDA | C – Pp | 8.70 | 2.64 | 3.297 | **0.0068** |
|  | C – Rs | 27.56 | 2.52 | 10.939 | **<.0001** |
|  | C – Sy | 31.15 | 2.38 | 13.073 | **<.0001** |
|  | Pp – Rs | 18.86 | 2.69 | 6.999 | **<.0001** |
|  | Pp – Sy | 22.45 | 2.57 | 8.744 | **<.0001** |
|  | Rs - Sy | 3.59 | 2.44 | 1.467 | 0.4605 |
| CAT | C – Pp | 2.124 | 0.520 | 4.089 | **0.0004** |
|  | C – Rs | 0.310 | 0.496 | 0.624 | 0.9242 |
|  | C – Sy | -0.332 | 0.469 | -0.707 | 0.8940 |
|  | Pp – Rs | -1.815 | 0.531 | -3.420 | **0.0046** |
|  | Pp – Sy | 2.456 | 0.505 | -4.859 | **<.0001** |
|  | Rs - Sy | -0.641 | 0.481 | -1.333 | 0.5438 |
| GST | C – Pp | 13.61 | 1.84 | 7.408 | **<.0001** |
|  | C – Rs | 35.77 | 1.75 | 20.397 | **<.0001** |
|  | C – Sy | 12.54 | 1.66 | 7.560 | **<.0001** |
|  | Pp – Rs | 22.17 | 1.88 | 11.817 | **<.0001** |
|  | Pp – Sy | -1.07 | 1.79 | -0.599 | 0.9323 |
|  | Rs - Sy | -23.24 | 1.70 | -13.656 | **<.0001** |

Table S11. Pairwise Comparisons for Microorganism for different parameters.

| Parameter | Contrast | p.value |  | Parameter | Contrast | p.value |
| --- | --- | --- | --- | --- | --- | --- |
| CHL | C – Pp | 1 |  | TPC | C – Pp | \|  \| \| --- \|  \| 0.1607 \| \| --- \| |
|  | C – Rs | 1 |  |  | C – Rs | **3.5e-06** |
|  | C – Sy | 1 |  |  | C – Sy | **0.0045** |
|  | Pp – Rs | 0.89 |  |  | Pp – Rs | **0.0326** |
|  | Pp – Sy | 1 |  |  | Pp – Sy | 1.0000 |
|  | Rs - Sy | 1 |  |  | Rs - Sy | **0.0271** |
| TFC | C – Pp | 0.24920 |  | APX | C – Pp | 0.2069 |
|  | C – Rs | **0.00060** |  |  | C – Rs | **0.0065** |
|  | C – Sy | 1 |  |  | C – Sy | **0.0064** |
|  | Pp – Rs | **0.0001** |  |  | Pp – Rs | 0.4555 |
|  | Pp – Sy | 1 |  |  | Pp – Sy | 1 |
|  | Rs - Sy | **0.0084** |  |  | Rs - Sy | 0.6205 |
| POX | C – Pp | 0.6369 |  | GR | C – Pp | 0.1570 |
|  | C – Rs | 0.4482 |  |  | C – Rs | **0.0084** |
|  | C – Sy | 0.4667 |  |  | C – Sy | **0.0135** |
|  | Pp – Rs | 1 |  |  | Pp – Rs | 0.5152 |
|  | Pp – Sy | 0.2327 |  |  | Pp – Sy | 1 |
|  | Rs - Sy | **0.0059** |  |  | Rs - Sy | 1 |
| SOD | C – Pp | 1 |  | CAR | C – Pp | 1 |
|  | C – Rs | 1 |  |  | C – Rs | 1 |
|  | C – Sy | 1 |  |  | C – Sy | 1 |
|  | Pp – Rs | 1 |  |  | Pp – Rs | 1 |
|  | Pp – Sy | 1 |  |  | Pp – Sy | 1 |
|  | Rs - Sy | 0.84 |  |  | Rs - Sy | 0.88 |
| SS | C – Pp | 1 |  |  |  |  |
|  | C – Rs | 1 |  |  |  |  |
|  | C – Sy | 0.26 |  |  |  |  |
|  | Pp – Rs | 1 |  |  |  |  |
|  | Pp – Sy | 1 |  |  |  |  |
|  | Rs - Sy | 1 |  |  |  |  |

**Table S12**. Levene’s Test for Homogeneity of Variances for different parameters (for Pollutant, including untreated plants) with Df value 33.

| Parameter | F value | Pr(>F) |
| --- | --- | --- |
| Shoot growth | 0.8483 | 0.703 |
| Root length | 0.8278 | 0.732 |
| MDA | 1.4284 | 0.07976 |
| CHL | 3.585 | 6.418e-08 *** |
| TPC | 2.1454 | 0.001116 ** |
| TFC | 1.702 | 0.01769 * |
| APX | 1.6813 | 0.01996 * |
| CAT | 0.9606 | 0.5355 |
| POX | 2.3975 | 0.0002102 *** |
| GR | 1.6529 | 0.02351 * |
| GST | 5.3172 | 8.409e-13 *** |
| SOD | 1.9553 | 0.003779 ** |
| CAR | 3.014 | 3.161e-06 *** |
| SS | 2.2574 | 0.0005347 *** |

**Table S13**. Tukey Post Hoc Test Results (Pollutant) for different parameters.

| Parameter | Comparison | p-value |
| --- | --- | --- |
| Shoot growth | C – PHE | 0.1372636 |
|  | C – PYR | 0.4376987 |
|  | C – NAPH | 0.7125935 |
|  | C – FLUO | 0.8764687 |
|  | NAPH - FLUO | **0.0225475** |
|  | FLUO - PHE | **0.0001956** |
|  | PHE – NAPH | 0.5513834 |
|  | PYR – NAPH | 0.9725026 |
|  | PYR – FLUO | **0.0037320** |
|  | PYR - PHE | 0.8907270 |
| Root Length | C – PHE | **0.0435227** |
|  | C – PYR | 0.4835141 |
|  | C – NAPH | 0.9504010 |
|  | C – FLUO | 0.6817805 |
|  | NAPH - FLUO | **0.0433872** |
|  | FLUO - PHE | 0.2567442 |
|  | PHE – NAPH | **0.0000331** |
|  | PYR – NAPH | **0.0115231** |
|  | PYR – FLUO | 0.9940071 |
|  | PYR - PHE | 0.4666443 |
| MDA | C – PHE | **0.0000343** |
|  | C – PYR | 0.9936713 |
|  | C – NAPH | 0.2170028 |
|  | C – FLUO | 0.9999008 |
|  | NAPH - FLUO | 0.0585430 |
|  | FLUO - PHE | **0.0000000** |
|  | PHE – NAPH | **0.0021065** |
|  | PYR – NAPH | **0.0051793** |
|  | PYR – FLUO | 0.9403745 |
|  | PYR - PHE | **0.0000000** |
| CAT | C – PHE | 0.5536535 |
|  | C – PYR | 0.9698607 |
|  | C – NAPH | 0.8277793 |
|  | C – FLUO | **0.0381929** |
|  | NAPH - FLUO | 0.0917680 |
|  | FLUO - PHE | 0.3977604 |
|  | PHE – NAPH | 0.9638845 |
|  | PYR – NAPH | 0.9769347 |
|  | PYR – FLUO | **0.0215630** |
|  | PYR - PHE | 0.7415387 |

**Table S14.** Pairwise Wilcoxon Test Results for different parameters (Pollutant).

| Parameter | Comparison | p-value |
| --- | --- | --- |
| CHL | C – PHE | **0.00094** |
|  | C – PYR | **0.00024** |
|  | C – NAPH | **0.00094** |
|  | C – FLUO | **0.00022** |
|  | NAPH - FLUO | **0.09657** |
|  | FLUO - PHE | 0.30240 |
|  | PHE – NAPH | 0.66527 |
|  | PYR – NAPH | 0.28934 |
|  | PYR – FLUO | 0.93715 |
|  | PYR - PHE | 0.45431 |
| TPC | C – PHE | **0.048** |
|  | C – PYR | 0.210 |
|  | C – NAPH | 0.310 |
|  | C – FLUO | 0.698 |
|  | NAPH - FLUO | 0.387 |
|  | FLUO - PHE | **0.048** |
|  | PHE – NAPH | 0.110 |
|  | PYR – NAPH | 0.564 |
|  | PYR – FLUO | 0.257 |
|  | PYR - PHE | 0.310 |
| TFC | C – PHE | 0.70 |
|  | C – PYR | 0.26 |
|  | C – NAPH | 0.33 |
|  | C – FLUO | 0.26 |
|  | NAPH - FLUO | 0.75 |
|  | FLUO - PHE | 0.26 |
|  | PHE – NAPH | 0.26 |
|  | PYR – NAPH | 0.75 |
|  | PYR – FLUO | 0.97 |
|  | PYR - PHE | 0.26 |
| APX | C – PHE | 0.317 |
|  | C – PYR | 0.331 |
|  | C – NAPH | 0.162 |
|  | C – FLUO | 0.058 |
|  | NAPH - FLUO | 0.306 |
|  | FLUO - PHE | 0.058 |
|  | PHE – NAPH | 0.306 |
|  | PYR – NAPH | 0.277 |
|  | PYR – FLUO | 0.058 |
|  | PYR - PHE | 0.953 |
| POX | C – PHE | 0.77 |
|  | C – PYR | 0.58 |
|  | C – NAPH | 0.36 |
|  | C – FLUO | 0.12 |
|  | NAPH - FLUO | 0.36 |
|  | FLUO - PHE | 0.12 |
|  | PHE – NAPH | 0.44 |
|  | PYR – NAPH | 0.44 |
|  | PYR – FLUO | 0.12 |
|  | PYR - PHE | 0.99 |
| GR | C – PHE | 0.43 |
|  | C – PYR | 0.25 |
|  | C – NAPH | 0.09 |
|  | C – FLUO | 0.09 |
|  | NAPH - FLUO | 0.85 |
|  | FLUO - PHE | 0.18 |
|  | PHE – NAPH | 0.09 |
|  | PYR – NAPH | 0.18 |
|  | PYR – FLUO | 0.32 |
|  | PYR - PHE | 0.85 |
| GST | C – PHE | **1.1e-12** |
|  | C – PYR | **4.5e-13** |
|  | C – NAPH | **4.5e-13** |
|  | C – FLUO | **5.6e-13** |
|  | NAPH - FLUO | 0.98 |
|  | FLUO - PHE | 0.98 |
|  | PHE – NAPH | 0.98 |
|  | PYR – NAPH | 0.12 |
|  | PYR – FLUO | 0.13 |
|  | PYR - PHE | 0.19 |
| SOD | C – PHE | 0.311 |
|  | C – PYR | **0.026** |
|  | C – NAPH | 0.221 |
|  | C – FLUO | 0.221 |
|  | NAPH - FLUO | 0.511 |
|  | FLUO - PHE | 0.302 |
|  | PHE – NAPH | 0.355 |
|  | PYR – NAPH | 0.185 |
|  | PYR – FLUO | 0.221 |
|  | PYR - PHE | **0.026** |
| CAR | C – PHE | 0.48134 |
|  | C – PYR | 0.22479 |
|  | C – NAPH | 0.48724 |
|  | C – FLUO | 0.05274 |
|  | NAPH - FLUO | **0.00013** |
|  | FLUO - PHE | **0.00097** |
|  | PHE – NAPH | 0.64564 |
|  | PYR – NAPH | **0.00950** |
|  | PYR – FLUO | 0.24172 |
|  | PYR - PHE | **0.00950** |
| SS | C – PHE | 0.9035 |
|  | C – PYR | **0.0075** |
|  | C – NAPH | 0.9035 |
|  | C – FLUO | 0.2983 |
|  | NAPH - FLUO | 0.2813 |
|  | FLUO - PHE | 0.2891 |
|  | PHE – NAPH | 0.9035 |
|  | PYR – NAPH | **0.0075** |
|  | PYR – FLUO | 0.2813 |
|  | PYR - PHE | **0.0057** |
